# Chemical Catalysis Guides Structural Identification for the Major *In Vivo* Metabolite of the BET Inhibitor JQ1

**DOI:** 10.1021/acsmedchemlett.3c00464

**Published:** 2024-01-02

**Authors:** Secondra Holmes, Prashi Jain, Kenneth Guzman Rodriguez, Jade Williams, Zhifeng Yu, Christian Cerda-Smith, Errol L. G. Samuel, James Campbell, John Michael Hakenjos, Diana Monsivais, Feng Li, Srinivas Chamakuri, Martin M. Matzuk, Conrad Santini, Kevin R. MacKenzie, Damian W. Young

**Affiliations:** † Center for Drug Discovery, Department of Pathology & Immunology, Baylor College of Medicine, Houston, Texas 77030, United States; ‡ Verna and Marrs McLean Department of Biochemistry and Molecular Pharmacology, Baylor College of Medicine, Houston, Texas 77030, United States

**Keywords:** Deuterated compounds, JQ1, Metabolic stability

## Abstract

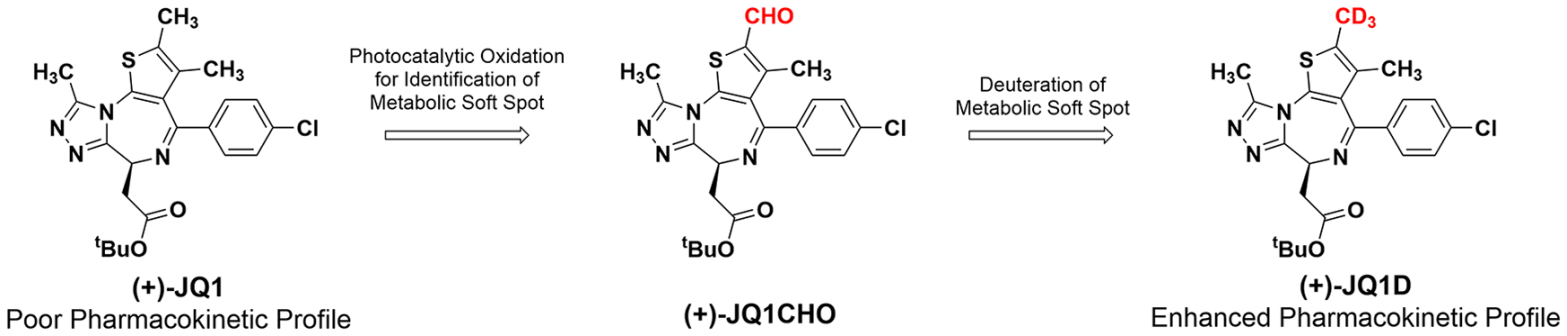

The bromodomain inhibitor (+)-JQ1 is a highly validated chemical probe; however, it exhibits poor *in vivo* pharmacokinetics. To guide efforts toward improving its pharmacological properties, we identified the (+)-JQ1 primary metabolite using chemical catalysis methods. Treatment of (+)-JQ1 with tetrabutylammonium decatungstate under photochemical conditions resulted in selective formation of an aldehyde at the 2-position of the thiophene ring [(+)-JQ1-CHO], which was further reduced to the 2-hydroxymethyl analog [(+)-JQ1-OH]. Comparative LC/MS analysis of (+)-JQ1-OH to the product obtained from liver microsomes suggested (+)-JQ1-OH as the major metabolite of (+)-JQ1. The 2-thienyl position was then substituted to generate a trideuterated (−CD_3_, (+)-JQ1-D) analog having half-lives that were 1.8- and 2.8-fold longer in mouse and human liver microsomes, respectively. This result unambiguously confirmed (+)-JQ1-OH as the major metabolite of (+)-JQ1. These studies demonstrate an efficient process for studying drug metabolism and identifying the metabolic soft spots of bioactive compounds.

Members of the bromodomain and extra-terminal domain (BET) family of proteins (in humans: BRD2, BRD3, BRD4, and BRDT) play key roles in regulating gene transcription through interactions with chromatin during cellular proliferation and differentiation and are implicated in latent viral infection of host cells and in oncogenesis.^1−3^ Two tandem bromodomains present in BET proteins both bind acetylated lysine residues and act as chromatin-targeting modules that decipher the histone acetylation code.^4^ The discovery of (+)-JQ1 (**1**), a cell-permeable small molecule that binds to BRD4 with high potency and selectivity, established that small molecules could target protein–protein interactions made by epigenetic readers.

In a previous report,^5^ our laboratory showed that (+)-JQ1 also blocks histone acetyllysine binding by bromodomain testis-specific protein (BRDT), which is essential for chromatin remodeling during spermatogenesis. These studies employing (+)-JQ1 validated BRDT as a target for reversible, nonhormonal male contraception. Other studies using (+)-JQ1 have demonstrated the efficacy of disrupting BET family protein interactions in hematological malignancies, glioblastoma, medulloblastoma, hepatocellular carcinoma, colon cancer, pancreatic cancer, prostate cancer, lung cancer, and breast cancer.^6,7^ (+)-JQ1 is thus an attractive tool compound for probing the underlying biology of the bromodomain and BET family proteins.

As a prominent chemical probe, (+)-JQ1 is potent, reasonably selective, has good cell permeability and high-affinity target engagement, and its enantiomer is an excellent inactive control. However, the short *in vivo* half-life (about an hour^8^) has limited utility. A recent metabolism study of (+)-JQ1 by us showed nine different (+)-JQ1 metabolites in human and mouse liver microsomes.^9^ The major metabolite was formed in human liver microsomes (HLM) and mouse liver microsomes (MLM) at yields of 63% and 79%, respectively. Thus, modifications to slow the production of this leading metabolite would enhance the *in vivo* half-life of (+)-JQ1. Synthesis of a more stable analogue of (+)-JQ1 would in turn be enabled by precise identification of the pronounced metabolite. Based on LC/MS, the major metabolite was postulated to be monohydroxylated on the thienotriazolodiazepine core of (+)-JQ1. Unfortunately, the precise site of this major hydroxylation event could not be determined by MS fragmentation, leading only to speculation that it could occur at one of four possible sites around the thienotriazolodiazepine scaffold.^9^ Scaling up the production of metabolites using microsomes is an impractical method due to challenges associated with the small scale and limited capacity of microsomal reactions. The synthesis of putative drug metabolites followed by the comparison of their MS/MS fragmentation patterns with those of metabolites produced in liver microsomes is a common route for metabolite identification; however, it can be laborious and time-consuming.^10−12^ Alternatively, substantial efforts are directed toward using computational approaches to predict compound susceptibility toward liver cytochrome P450 enzymes.^13,14^ Computational methods may narrow the field of possible metabolites, but total syntheses are generally still needed for structural confirmation.

We envisioned an alternative approach for studying and mitigating drug metabolism by using chemical catalysis methods that might mimic the actions of cytochrome P450 enzymes on drugs.^15^ Catalysts which display analogous reactivity to cytochrome P450 enzymes could potentially produce drug “metabolites” or analogs at metabolically reactive positions directly from the drug substance in sufficient quantity for structure determination by structure-based experiments such as NMR. Obtaining putative metabolites of drugs and biologically important small molecules would obviate the need for a total synthesis to elucidate a metabolite’s structure. Herein we describe the identification of the major metabolite of (+)-JQ1 by using a chemical-catalysis-based approach. We further detail how knowledge of the major metabolite was exploited to generate (+)-JQ1 analogs with improved metabolism.

The major (+)-JQ1 metabolite is generated by several human cytochrome P450 enzymes but primarily by CYP3A4.^9^ We previously reported that human and mouse liver microsomes produce monohydroxylated (+)-JQ1 as a major metabolite, and MS–MS data showed that the hydroxylation site might be the triazole, thiophene, or diazepine heterocycle.^9^ These data, however, could not resolve which of these heterocycles served as the primary reactive site. To provide insight into which site might be the source of the major metabolite, we used the online software tools SMARTCyp^16,17^ and SOMP^18^ to predict the relative reactivities of (+)-JQ1 sites with CYP3A4 (Figure 1). SMARTCyp predicted the chiral carbon to be most reactive (score = 44) followed by the thiophene 2-methyl (50), the triazole methyl (51), and thiophene 3-methyl (51). Alternatively, SOMP predicted the triazole methyl to be most reactive (score = 0.833), followed by the chiral carbon (0.364), the thiophene 2-methyl (0.336), the thiophene sulfur (0.161), and the thiophene 3-methyl (0.159). SOMP also predicts the possible metabolites for CYP2C19, which is the second-most-active enzyme generating M1.^9^ For CYP2C19, SOMP ranked the triazole methyl highest (0.77), followed by the chiral carbon (0.482), the thiophene sulfur (0.296), the thiophene 3-methyl (0.284), the *tert*-butyl carbons (0.098), and the thiophene 2-methyl (0.034). Lacking a consensus in these metabolite predictions, we sought chemical catalytic oxidative methods that would enable us to identify reactive sites and perhaps also provide a means of introducing functional groups that could increase metabolic stability.

**Figure 1:**
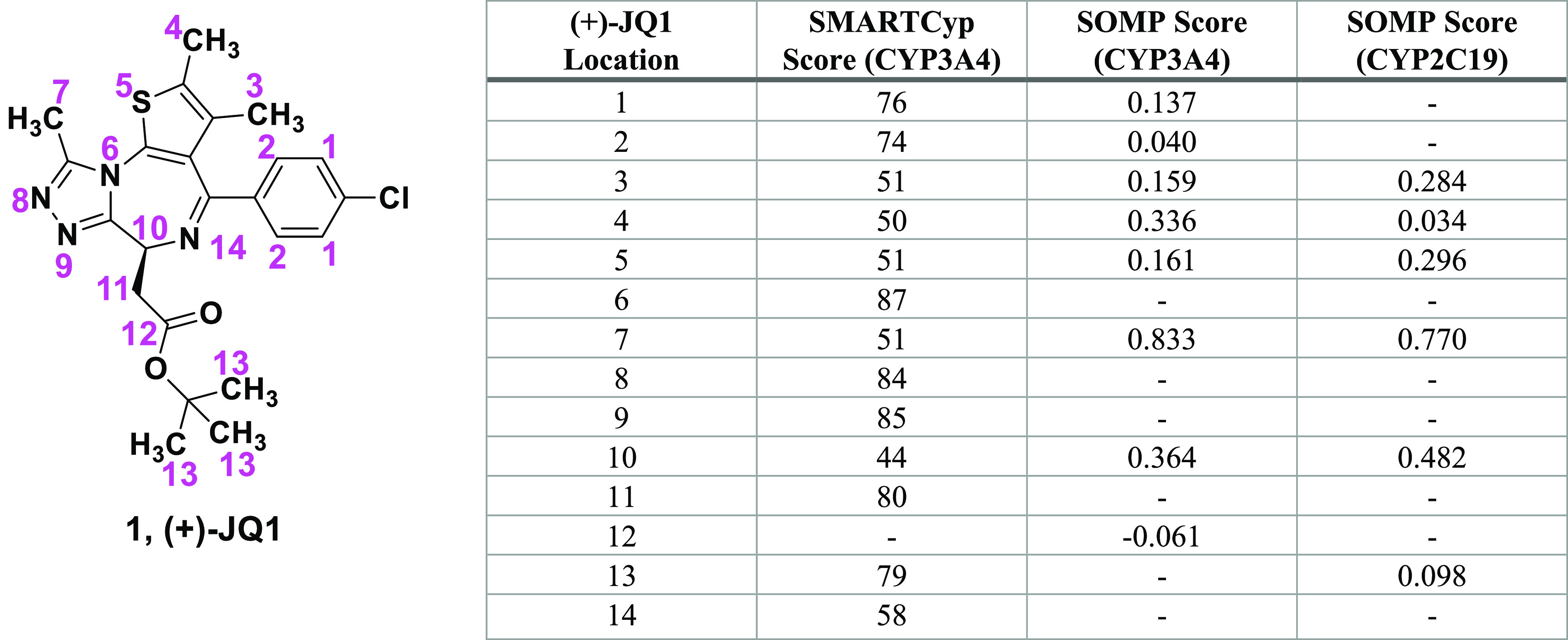
Metabolic site prediction scores, as determined *in silico* by the SMARTCyp and SOMP programs. Sites with a higher probability of being metabolized result in lower scores using the SMARTCyp algorithm and higher scores with the SOMP algorithm.

The tetrabutylammonium salt of decatungstate anion [W_10_O_32_]^4–^ (TBADT, **2**) (Figure 2a) is a well-characterized polyoxometalate catalyst which is widely used to promote the direct functionalization of unactivated sp^3^ C–H bonds using light irradiation.^15,19,20^ TBADT photocatalysis is attractive because of its mild reaction conditions, inexpensive cost, broad substrate scope, and functional group tolerance.^21^ Britton and co-workers discovered the direct fluorination at unactivated sp^3^ C–H bonds in the presence of TBADT and *N*-fluorobenzenesulfonimide (NFSI).^19^ The authors demonstrated impressive substrate site selectivity for fluorination and hypothesized that it might derive from a preference for the most labile C–H bond. The site of TBADT-mediated fluorination could also selectively align with sites in drug compounds that are susceptible to P450-mediated metabolism because that also predictably occurs at the labile, easily oxidizable positions. If TBADT showed similar chemical reactivity with (+)-JQ1 as the liver microsome cytochrome P450 oxidases (CYPs), then the TBADT-directed fluorination events should occur at the same site as the biological hydroxylation. We applied this photocatalytic methodology to (+)-JQ1 as denoted in Figure 2b. Irradiation of (+)-JQ1 (4 h, 365 nm) in the presence of 2 mol % TBADT and 1.5 equiv of NFSI in dry acetonitrile afforded almost no conversion of (+)-JQ1 to (+)-JQ1-F (**3**) (by LC/MS). Under an inert atmosphere and high catalyst loading, only a trace amount of a monofluorinated product was produced. Our attempts to optimize the photochemical fluorination reaction of (+)-JQ1 included many variations of the reaction reagent concentrations and prolonged reaction times that were periodically monitored to no avail, but with careful monitoring we noticed the consistent formation of a small amount of oxidized product with *m*/*z* 470.2 [M + H]^+^. This result was congruent with aldehyde formation ((+)-JQ1 + O – 2H, (+)-JQ1-CHO (**4**)), so we pivoted to the possibility of using TBADT for selective oxidation.^22^ Irradiation of (+)-JQ1 (4 h, 365 nm) with 2% TBADT in acetonitrile under open air produced a major product (20%) with *m*/*z* 470.2, which matches a minor metabolite product obtained by oxidation of a (+)-JQ1 methyl group to an aldehyde in our *in vitro* MLM/HLM metabolic studies.^9^ Serial recharges of TBADT and repeated irradiation (20 h total) resulted in 90% conversion to (+)-JQ1-CHO. We purified the product by TLC to recover a 44% yield, performed analysis by NMR (Figure S1), and confirmed that the oxidation had occurred at the thiophene 2-position ((+)-JQ1-CHO; Figure 2c).

**Figure 2:**
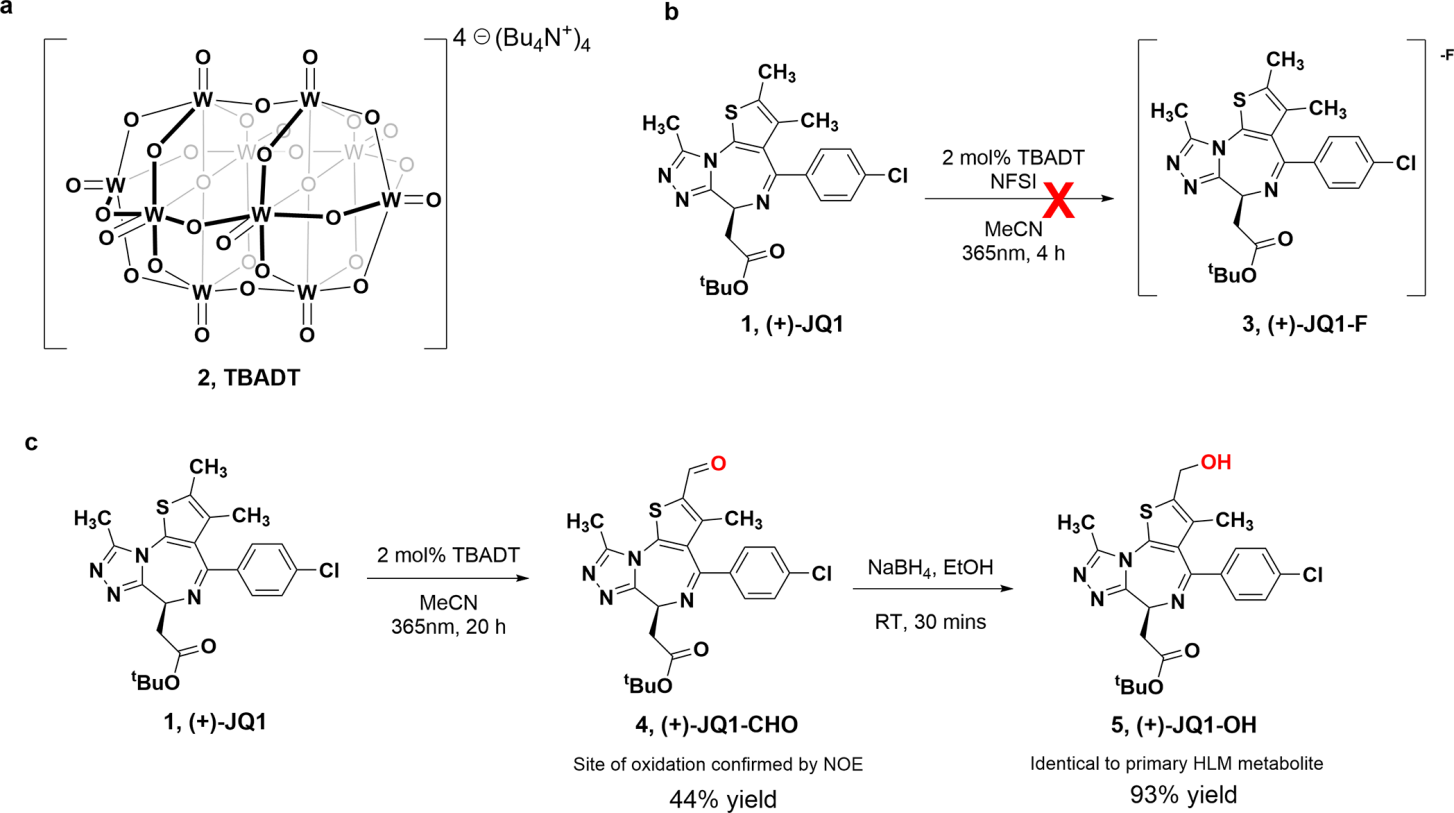
Photocatalytic oxidation of JQ1. (a) Molecular structure of TBADT. (b) TBADT-promoted oxidative fluorination. (c) TBADT-promoted site-selective oxidation of (+)-JQ1.

Since the putative major metabolite, M1, was predicted to be an alcohol (*m*/*z* 473), we treated (+)-JQ1-CHO with NaBH_4_ to afford the alcohol (+)-JQ1-OH (**5**) (Figure 2c). An identical retention time and equal exact mass were observed for (+)-JQ1-OH as for M1. In our previous study, analysis of the fragmentation pattern led to an inconclusive structural determination of an oxidation product. Based on the LC/MS of the 2-hydroxylated compound, (+)-JQ1-OH, we were able to match the fragmentation pattern of the alcohol to the pattern of the major phase I (+)-JQ1 metabolite. We note that without MS fragmentation of the thienotriazolodiazepine core or knowing the LC retention times of the other possible oxidation products, we cannot unequivocally rule them out. Nonetheless, the correspondence between the microsomal metabolite and alcohol (+)-JQ1-OH supports our hypothesis that TBADT and other catalysts can mimic, in a selective manner, the oxidative metabolism of complex drug-like small molecules, being explored at later stages of drug discovery.

Having identified the potential major site of metabolism, we next turned our attention toward modifying this site for the production of more metabolically stable analogs. Given the unsuccessful attempts to introduce fluorine through the TBDAT-mediated fluorination reaction, we decided to pursue DeoxoFluor-mediated fluorination on the (+)-JQ1 oxidative products (+)-JQ1-CHO (**4**) and (+)-JQ1-OH (**5**). Exposure of (+)-JQ1-CHO to DeoxoFluor (3.5 equiv, DCM, RT, 48 h) afforded the difluoromethyl (+)-JQ1 analog (+)-JQ1-F_2_ (**6**) in 34% isolated yield. Similarly, treating (+)-JQ1-OH with DeoxoFluor (2 equiv, DCM, RT, 24 h) provided the fluoromethyl (+)-JQ1 analogue (+)-JQ1-F_1_ (**7**) in 38% isolated yield (Figure 3). Although (+)-JQ1-F_2_ shows a substantially improved half-life in human liver microsomes (0.1 mg/mL microsomal protein), both (+)-JQ1-F_1_ and (+)-JQ1-F_2_ lost potency against BRDT (Table 1). The loss of potency (Table 1 and Figure S13) for these (+)-JQ1 fluorinated analogs led us to abandon the semisynthesis of a trifluorinated (+)-JQ1 analog. Using the selectivity of photochemical oxidation to first identify the possible site of metabolism and then direct its rational modification allowed us to bypass many steps involved in the complete synthesis of these analogs. This method could potentially be applicable to other drug-like compounds.^15,23−25^

**Table 1:** Human Liver Microsomal *t*_1/2_ and BRDT Binding Data for JQ1 and Fluorinated Analogs (0.1 mg/mL Microsomal Protein)

	*t*_1/2_ (min)	K_d_ (nM)
(+)-JQ1	49	188
(+)-JQ1-F_1_	52	722
(+)-JQ1-F_2_	125	3920

**Figure 3:**
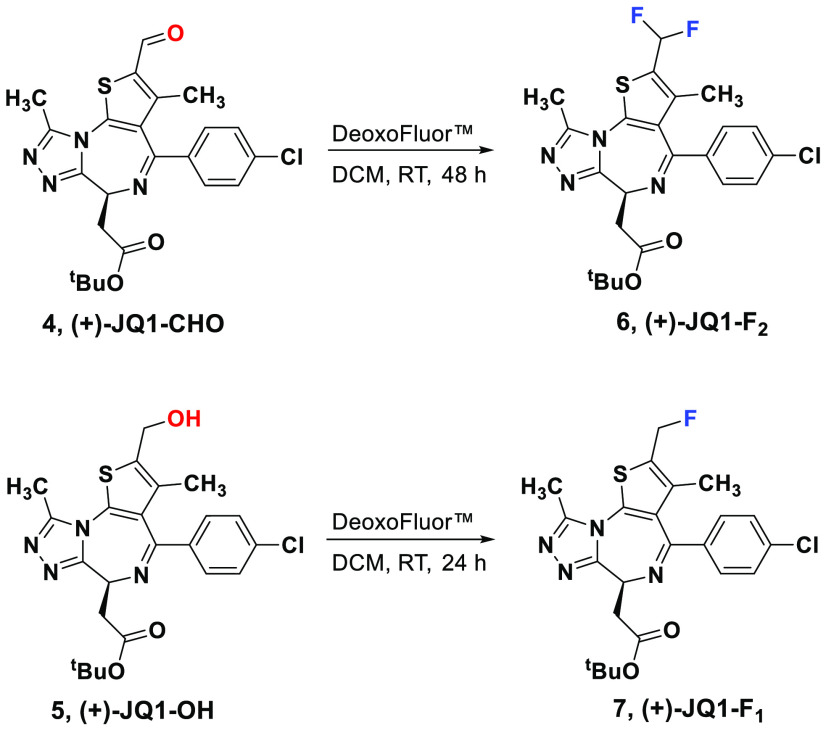
Synthesis of (+)-JQ1-F_2_ and (+)-JQ1-F_1_.

Because fluorine substitution for hydrogen on the thiophene 2-methyl adversely affects potency (Table 1), we sought to modify (+)-JQ1 isosterically by trideuterating the reactive thiophene 2-methyl group. Deuterium and hydrogen make bonds of nearly identical bond lengths, making them nearly perfectly isosteric, so deuteration may decrease metabolism due to the primary kinetic isotope effect with no impact on compound affinity to targets. Deuterated molecules have shown utility in the study of reaction mechanisms, elucidation of biosynthetic pathways, and enhancement of drug metabolic stability.^26−30^ Deuterated analogs, which are regarded as a new chemical entity, are in clinical trials,^31^ and the first-ever FDA-approved deuterated drug deutetrabenazine in 2017^32^ is twice as stable as the protiated version. Although chemical methods for the late-stage introduction of deuterium are becoming available,^33^ we undertook the *de novo* synthesis of the enantiomerically pure 2-trideuteriomethyl analog of (+)-JQ1, (+)-JQ1-D (**14**) (Figure 4).

**Figure 4:**
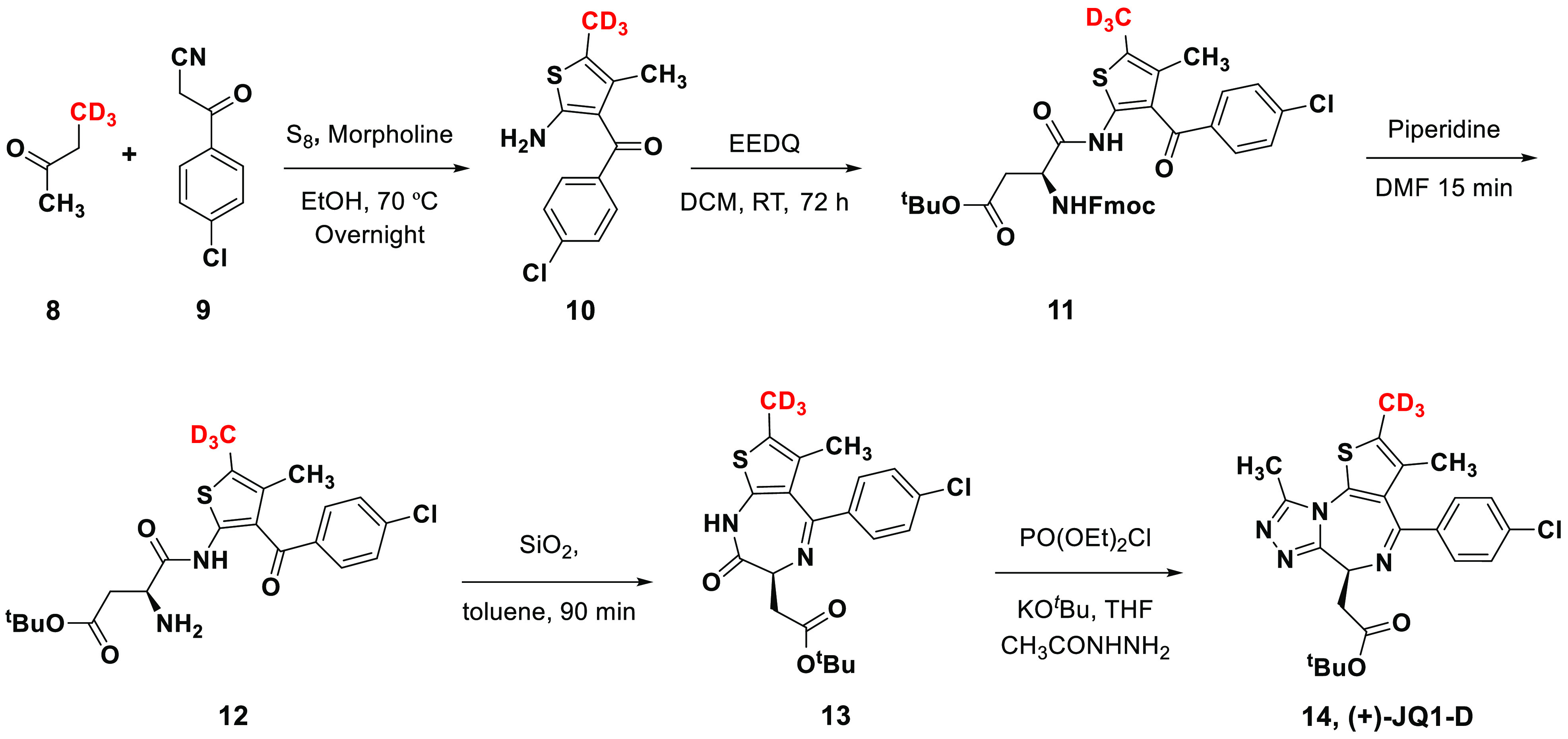
Synthesis of (+)-JQ1-D.

Based on the previously reported (+)-JQ1 synthesis,^8^ the route initiated with a Gewald reaction of 4,4,4-trideutero-2-butanone (**8**) with 3-(4-chlorophenyl)-3-oxopropionitrile (**9**) in the presence of elemental sulfur and morpholine to produce thiophene **10** trideuterated at the 2-position (Figure 4). We confirmed the −CD_3_ position on the thiophene ring using 2D NMR (Figure S14–S17). The substituted thiophene ring was relatively unstable at room temperature and was thus directly coupled with a differentially protected aspartic acid to provide compound **11**. The previously reported PyBOP coupling conditions led to extensive racemization (60:40 er) at C2 of the aspartate with a poor yield in our hands. In order to maintain the stereochemical integrity as well as improve the overall yield, we tested different amide coupling conditions and achieved success with *N*-ethoxycarbonyl-2-ethoxy-1,2-dihydroquinoline (EEDQ). The EEDQ reagent in DCM at room temperature for 4 days provided 75–80% yield with minimal racemization (95:5 er). Deprotection of the Fmoc group provided compound **12**, which upon subjection to silica in toluene underwent cyclization to deliver compound **13**. The final triazole ring formation to give (+)-JQ1-D was effected by the reaction of compound **13** with diethyl phosphorochloridate and acetohydrazine under basic conditions. The synthesized (+)-JQ1-D was analyzed using chiral HPLC and found to have an enantiomeric ratio of 95:5. We subjected this material to preparative chiral column chromatography using CHIRALPAK ID (DIACEL) to achieve (+)-JQ1-D with >99% ee for our further studies and to isolate pure (−)-JQ1-D as a negative control.

The binding affinities of (+)-JQ1-D against BRD4 and BRDT were measured with an Amplified Luminescent Proximity Homogeneous Assay Screen (ALPHAScreen). Histidine-tagged bromodomain-containing protein constructs of BRD4 and BRDT were used to form complexes with a biotin-tagged (+)-JQ1 probe.^34^ As anticipated, based on this competitive binding assay, (+)-JQ1-D affinity for BRD4 and BRDT (53 nM for BRD4 and 103 nM for BRDT) is similar to that of (+)-JQ1 (49 nM for BRD4 and 124 nM for BRDT) (Figure 5).

**Figure 5:**
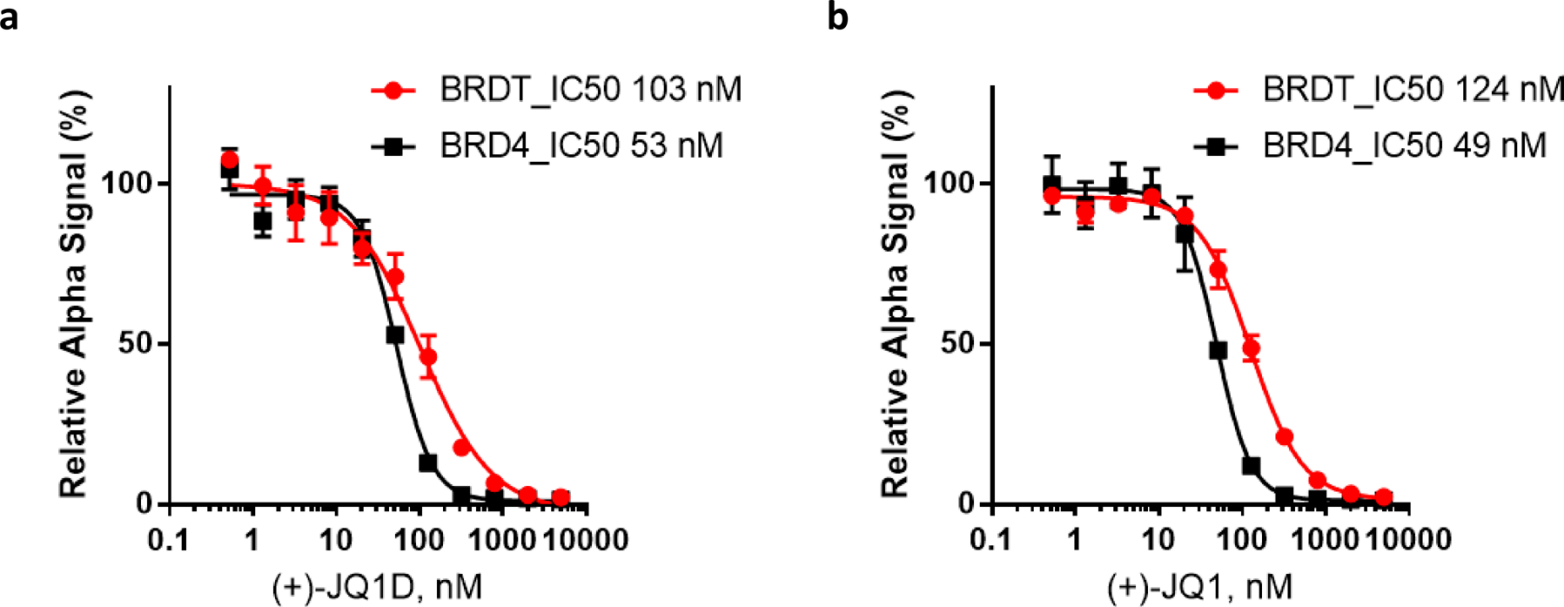
ALPHAScreen assay results for the IC_50_ determination of (a) (+)-JQ1-D and (b) (+)-JQ1.

Next, to evaluate the metabolic stability of (+)-JQ1-D, we subjected 1:1 mixtures of (+)-JQ1 and (+)-JQ1-D to either mouse or human liver microsomes (0.5 mg/mL microsomal protein) and analyzed the products by LC/MS to determine the isotopic effect on *in vitro* P450-mediated metabolism. The major metabolite generated from (+)-JQ1-D coelutes with that generated from (+)-JQ1, and its exact mass indicates the presence of two deuterium atoms, consistent with oxidation at the thiophene 2-methyl position and loss of one deuterium. Given the precise location of the −CD_3_ group, this result unambiguously confirms our earlier conclusion that the 2-position on (+)-JQ1 is the primary site of CYP metabolism. Importantly, deuteration at the 2-methyl position increases the *in vitro* half-life in mouse (or human) liver microsomes 1.8-fold (or 2.8-fold), indicating a significant primary deuterium isotope effect on this reaction, especially in human liver microsomes (Table 2). These substantial increases in half-life could influence the total exposure to (+)-JQ1, as known in case of deuterated drugs.^32^

**Table 2:** Microsomal *t*_1/2_ (min) for (+)-JQ1 and (+)-JQ1-D (0.5 mg/mL Microsomal Protein)

	MLM	HLM
(+)-JQ1	11.0	7.9
(+)-JQ1-D	19.8	22.5
fold improvement	1.8	2.8

To test the effects of deuteration on total exposure, we administered a 1:1 mixture of (+)-JQ1 and (+)-JQ1-D to mice (male and female) intraperitoneally (50 mg/kg) and analyzed the pharmacokinetics of the compounds (Figure 6 and Table 3). Despite large differences in metabolism between individual mice (Figure 6), on average both (+)-JQ1 and (+)-JQ1-D are cleared more rapidly in female mice than in male mice, and total exposure (area under the curve, AUC_0–*t*_) in female mice is about half of the total exposure in male mice (Table 3). Deuteration of the 2-methyl position improves total exposure by essentially the same modest proportion in mice of either gender (by 23% in female mice and 25% in male mice) compared to (+)-JQ1 total exposure. The observed improvement in male mice was not determined to be significant, but the gender differences are not unprecedented in human drug metabolism, as the clearance of CYP3A substrates occurs more rapidly by females than males.^35^ Gender differences in hepatic CYP expression have been identified in mice, rats, and humans,^36^ including a female-specific CYP3A family member in mice,^37^ and circadian variations in mice can accentuate the sex differences in CYP3A isoform expression.^38^ We also observed an increase in AUC_0–*t*_ between (+)-JQ1 and (+)-JQ1-D as the half-life (*t*_1/2_) decreases. It is noteworthy that the relationship between AUC and half-life can be affected with no correlation due to influence on both by various factors such as target-mediated drug disposition, drug formulation, route of administration, metabolism, absorption, and elimination pathways.^39,40^ AUC_0–*t*_ represents the cumulative effect, while *t*_1/2_ represents only the rate at which the drug is eliminated from the body. (+)-JQ1-D caused a shorter *in vivot*_1/2_, indicating that it is eliminated from the body relatively quickly, but during the time it is present in the body it attains a higher peak concentration, which could be explained by its decreased metabolism (shown in the microsomal half-life study) or a higher rate of absorption.

**Figure 6:**
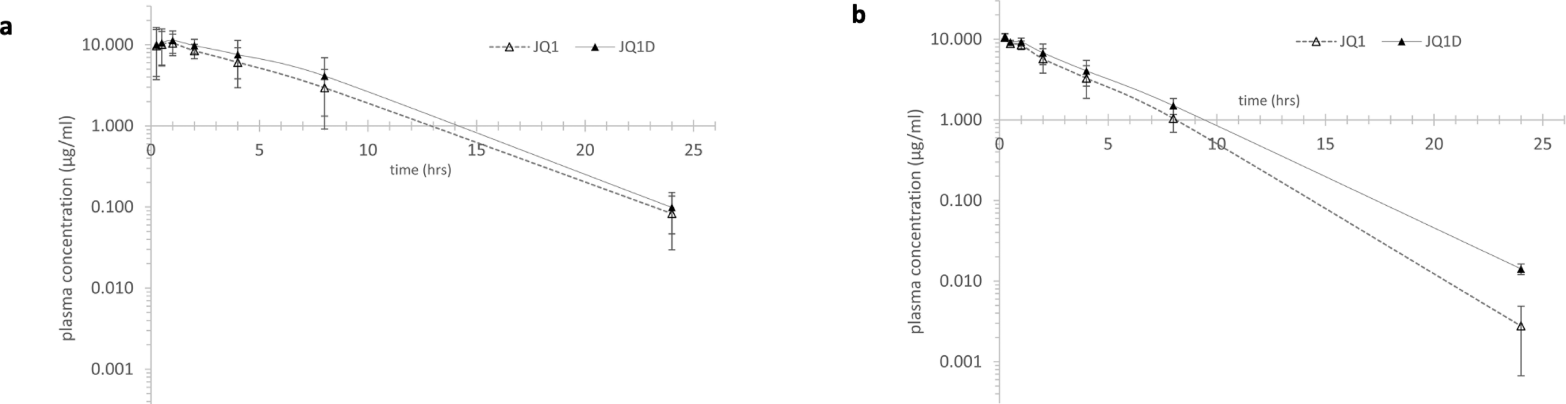
(+)-JQ1 and (+)-JQ1-D metabolism in liver microsomes of (a) male mice and (b) female mice.

**Table 3:** Pharmacokinetic Parameters for (+)-JQ1 and (+)-JQ1-D in Mice

	male (*n* = 4)		female (*n* = 4)	
	(+)-JQ1	(+)-JQ1-D	(+)-JQ1	(+)-JQ1-D
AUC_0–*t*_ (μg mL^–1^ h^–1^)	75.1	94.2	41.1	50.5
CL/*F*_obs_ (mg (μg/mL)^−1^ h^–1^)	0.661	0.528	1.22	0.989
*t*_1/2_ (h)	3.36	3.01	1.93	2.46

The large differences between individual mice in this small study may lead to questions about the significance of the modest average differences obtained from this pharmacokinetic analysis. Given that our main goal was to assess the relative effects of deuteration on clearance of (+)-JQ1 rather than determining the absolute pharmacokinetic parameters of (+)-JQ1 and (+)-JQ1-D, we examined the isotopomeric ratios for (+)-JQ1 and for its major metabolite M1^9^ from the pharmacokinetic time course for evidence of the effects of deuteration. In both male and female mice, the (+)-JQ1/(+)-JQ1-D ratio drops steadily over the examined time course (Figure 7), indicating that (+)-JQ1 is cleared more rapidly than (+)-JQ1-D.

**Figure 7:**
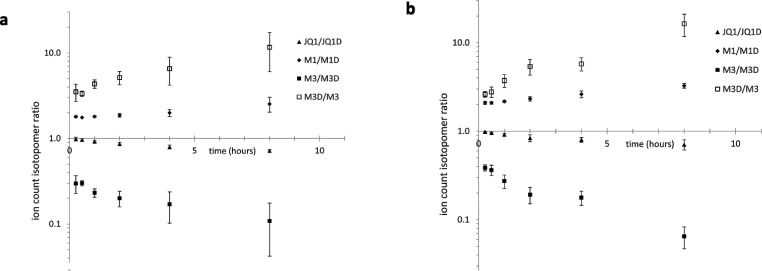
Isotopomeric ratios for (+)-JQ1 and metabolites in (a) male mice and (b) female mice.

Importantly, the variance in these ratios from mouse to mouse is very small: at the first time point (15 min), the isotopomeric ratios are 0.9869 ± 0.049 (*n* = 4 male) and 0.9828 ± 0.0158 (*n* = 4 female). Quantifying the isomer ion counts in the same sample from a single LC/MS trace eliminates many sources of error and uncertainty and makes it possible to track the relative effects of deuteration with high precision and accuracy.

Analyzing the isotopomeric ratios for the major metabolite M1, which corresponds to oxygenation of the thiophene 2-methyl, reveals M1/M1D ratios increase modestly over time (Figure 7). At the first time point, M1/M1D is 1.799 ± 0.043 in male mice and 2.095 ± 0.089 in female mice, and this is followed by a 1.8-fold (or 2.05-fold) slower production of M1D compared to M1. This isotopomeric analysis is consistent with the data in Table 2, which show a 1.8-fold effect of deuteration on the *in vitro* half-life (for pooled mouse liver microsomes).

Though produced in a drastically lower quantity than M1, M3 was identified as another major metabolite of JQ1, which MS–MS analysis indicates corresponds to oxygenation of the chlorophenyl ring.^9^ Remarkably, the production of M3 is strongly enhanced by deuteration (Figure 7). The M3D/M3 ratios at the second time point are 3.34 ± 0.23 in male mice and 2.78 ± 0.37 in female mice.

A single CYP may generate many products from one substrate. We previously showed that human CYP3A4 acts on (+)-JQ1 to produce the singly hydroxylated species M1 and M3 as well as other metabolites whose masses imply dihydroxylation and/or dehydrogenation.^9^ A diverse product profile may result from different substrate binding modes, but the intrinsic reactivity differences of moieties within the substrate will also affect the rate at which different products are generated. If a CYP that binds (+)-JQ1 can generate either M1 or M3, probably through different substrate binding modes, then having the deuterium kinetic isotope decrease the reactivity at the thiophene 2-methyl may increase the production of M3D (or some other product) rather than the substrate being released from the binding site without reaction. The 2-fold decrease in the rate of M1D production (relative to M1) and the 3-fold faster production of M3D (relative to M3) that we report here combine to give a 6-fold swing in relative rates that represents a substantial “switching” of product specificity, which has been seen before with deuterium kinetic isotope effects in CYP3A4-mediated hydroxylation reactions of testosterone.^41^ These studies highlight the flexibility that P450 enzymes can display to shunt to other reactivity modes when primary modes resulting from changing the drug’s parent structure are made less available.

Understanding metabolic pathways is often relegated to the late stages of drug discovery endeavors, primarily because molecular optimization tends to focus on binding and functional studies rather than ADME studies. The structural identification of drug metabolites is mostly based on LC/MS techniques; however, in some instances these methods preclude metabolite structural assignment. In this study, we present an example of using chemical catalysts to rapidly initiate the process of identifying metabolic sites, demonstrating their efficient integration into drug development. It was previously reported that the bromodomain inhibitor (+)-JQ1 produced a major metabolite that was thought to be an alcohol resulting from oxidation at an unidentified site. The reaction of (+)-JQ1 with a tungsten-based catalyst led to a single oxidized product, in which the 2-thienyl methyl group was converted to an aldehyde. By comparing the LC/MS profiles of the reduced product obtained from chemical catalysis to the results from liver microsome studies, we identified that the 2-methyl group was the likely site of (+)-JQ1 oxidation *in vivo*. Successful syntheses of (+)-JQ1-D having a trideuteromethyl group at the reactive site led to an increase of the microsomal half-life of (+)-JQ1-D and confirmed the 2-methyl group of (+)-JQ1 as the major site of metabolism. There was a negligible effect on substrate binding, and the pharmacokinetic profile improved. Surprisingly, deuteration significantly boosts the production of an alternate metabolite, M3. CYP3A4 acts on (+)-JQ1 to generate the singly hydroxylated M1 and M3 species, and as M1 production is decreased, a “switching” phenomenon occurs toward the production of M3. Taken together, our findings illuminate that a chemical reaction can be established, mirroring CYP reactivity in HLMs, on a fully intact drug molecule and that such information can be readily applied to generate a more metabolically stable analog. Given its efficiency, the chemical catalysis approach can be applied at much earlier stages of the drug discovery process, which has the potential to accelerate the development of new therapeutics.

## Supporting Information Available

Experimental information and NMR data for synthesized compounds (PDF)

## Abbreviations

BET: bromodomain and extra-terminal domain
BRDT: bromodomain testis-specific protein
TBADT: tetrabutylammonium decatungstate
NFSI: *N*-fluorobenzenesulfonimide
EEDQ: *N*-ethoxycarbonyl-2-ethoxy-1,2-dihydroquinoline
ALPHAScreen: Amplified Luminescent Proximity Homogeneous Assay Screen
HLM: human liver microsomes
MLM: mouse liver microsomes
AUC_0–*t*_: area under the curve
