# Supplementary material for: Chemical Catalysis Guides Structural Identification for the Major In Vivo Metabolite of the BET Inhibitor JQ1
Source: ACS Med Chem Lett. 2024 Jan 2;15(1):107–15. doi: 10.1021/acsmedchemlett.3c00464 (PMC10788937; doi:10.1021/acsmedchemlett.3c00464)

Chemical catalysis guides structural identification for the major in vivo metabolite of the BET inhibitor JQ1

Secondra Holmes,[a,b, ‡] Prashi Jain,[a,b, ‡] Kenneth Guzman Rodriguez,[a,b] Jade Williams,[a,b] Zhifeng Yu,[a,b] Christian Cerda-Smith,[a] Errol L. G. Samuel,[a] James Campbell,[a] John Michael Hakenjos,[a] Diana Monsivais,[a] Feng Li,[a] Srinivas Chamakuri,[a] Martin M. Matzuk,[a] Conrad Santini,[a] Kevin R. MacKenzie[a, b,*] and Damian W. Young[a,b,*]

AUTHOR ADDRESS.

[a] Center for Drug Discovery, Department of Pathology & Immunology, Baylor College of Medicine, Houston, Texas 77030 USA

[b] Verna and Marrs McLean Department of Biochemistry and Molecular Pharmacology, Baylor College of Medicine, Houston, Texas 77030 USA

Table of Contents

General Information......S1

Detailed Experimental Procedures......S2

NMR Data......S9

General Information

All chemicals, reagents, and instrument consumables were obtained from commercial sources without further modification or purification, unless noted otherwise. No unexpected or unusually high safety hazards were encountered. The HPLC-UV/MS system used for monitoring reactions consisted of a HPLC instrument coupled to a low resolution mass spectrometer with single quadrupole ionization operating in either positive or negative ion mode. The analytical method was equipped with a C18 column (2.1 × 50 mm, 1.8 mm) eluting with a linear gradient of 95%/5% water/CH3CN (modified with 0.05% formic acid; t = 0 min; flow = 0.35 mL/min) to 95%/5% CH3CN/water (t = 3.5 min; flow = 0.5 mL/min) then 95%/5% CH3CN/water to t = 5min (0.5 mL/min). Peak detection was done at 254 nm and 230 nm for UV active compounds. NMR spectra were obtained on Bruker AVANCE (600 MHz) or AVANCE (800 MHz) Systems. 1H NMR spectrum multiplicities are notated as: s (singlet), br (broad), d (doublet), t (triplet), q (quadruplet), m (multiplet). All compounds are >95% pure by HPLC analysis

Detailed Experimental Procedures

Catalyst preparation: The procedure was carried out on the same scale as described in the literature method[19] Tetrabutylammonium bromide (2.4 g) and sodium tungstate dihydrate (5.0 g) were each separately dissolved in 150 mL of deionized water in an Erlenmeyer flask and kept at 90 °C with vigorous stirring. Concentrated hydrochloric acid was added dropwise to both solutions in order to adjust the pH to 2, at which point the tungstate solution turned slightly greenish yellow in color. The two solutions were then mixed and maintained at 90 °C for 30 min with stirring. A white suspension resulted. The reaction was cooled and the precipitated TBADT was filtered on a Buchner funnel. The solid was washed with water and then dried under high vacuum for 3 hours. Crude TBADT was suspended in dichloromethane (20 mL of solvent per gram of solid) and stirred for 2 hours. Pure TBADT was separated from the yellow supernatant solution by filtration on a Buchner funnel and drying under vacuum.

Preparation of *tert*-butyl (*S*)-2-(4-(4-chlorophenyl)-2-formyl-3,9-dimethyl-6*H*-thieno[3,2-*f*][1,2,4]triazolo[4,3-*a*][1,4]diazepin-6-yl)acetate, (+)-JQ1-CHO (4): A one-dram borosilicate vial with stir bar was charged with a solution of *tert*-butyl (*R*)-2-(4-(4-chlorophenyl)-2,3,9-trimethyl-6*H*-thieno[3,2-*f*][1,2,4]triazolo[4,3-*a*][1,4]diazepin-6-yl)acetate (JQ1; 30 mg, 0.066 mmol) in dry acetonitrile (ACN; 400 µL). Tetrabutylammonium-decatungstate (TBADT; 4.4 mg, 1.3 µmol, 0.02 eq) was added. The mixture was placed atop a magnetic stir plate and positioned between two 365 nm lamps (Chauvet NV-F18 Blacklight). The vial was sealed with a rubber septum. An air-filled balloon, attached by tubing to a hypodermic needle, was positioned above the vial and the needle passed through the septum. The vial and lamps were wrapped with aluminum foil. With stirring, the mixture was irradiated for 4 h. The mixture was filtered through a cotton plug in a Pasteur pipette and the filtrate evaporated. The derived residue was re-dissolved in dry ACN (400 µL) and recharged with fresh TBADT as above. Irradiation was resumed for 4 h. The above-described sequence was repeated two more times, with a total irradiation time of 20 h. Filtration of the mixture followed by evaporation of the solvent gave a yellow solid which was purified by silica gel chromatography (pTLC; 500 µm; 1% triethylamine/EtOAc) to give the title compound as a yellow oil (JQ1-CHO, 13.5 mg, 44%). The reaction was monitored by HPLC-UV/MS using solutions composed of 745 µL of *i*PrOH and 5 µL of sample, then filtered

through a syringe filter. **^{1}H NMR (600 MHz, CDCl3)** δ 10.14 (s, 1H), 7.43-7.39 (m, 4H), 4.64 (dd, J = 8.0, 6.0 Hz, 1H), 3.63 – 3.52 (m, 2H), 2.76 (s, 3H), 2.20 (s, 3H), 1.53 (d, J = 0.9 Hz, 9H). **^{13}C NMR (151 MHz, CDCl3)** δ 181.8, 170.6, 162.4, 155.1, 150.1, 144.6, 142.7, 137.4, 131.2, 81.2, 68.2, 60.4, 54.1, 37.7, 28.2, 15.1, 12.0.

Preparation of *tert*-butyl (*S*)-2-(4-(4-chlorophenyl)-2-(hydroxymethyl)-3,9-dimethyl-6*H*-thieno[3,2-*f*][1,2,4]triazolo[4,3-*a*][1,4]diazepin-6-yl)acetate, (+)-JQ1-OH (5): A rt solution of JQ1-CHO (5 mg, 0.011 mMol) in absolute ethanol (1 mL) was treated with solid $NaBH_4$ (0.033 mMol). The mixture was stirred for 1 h. Water was added and the product was extracted using EtOAc. The organic phase was dried over sodium sulfate, filtered and evaporated to give the title compound as a white solid (4.7 mg, 93%). **^{1}H NMR (800 MHz, Chloroform-d)** δ 7.43 (d, 2H) 7.36 (d, 2H), 4.84-4.89 (m, 2H), 4.59-4.56 (m, 1H), 3.57 (m, 2H) 2.72 (s, 3H), 1.53 (s, 9H).

Preparation of *tert*-butyl (*S*)-2-(4-(4-chlorophenyl)-2-(difluoromethyl)-3,9-dimethyl-6*H*-thieno[3,2-*f*][1,2,4]triazolo[4,3-*a*][1,4]diazepin-6-yl)acetate (+)-JQ1-F_2 (6): A solution of (+)-JQ1-CHO (8.2 mg, 0.017 mmol) in dichloromethane (DCM; 0.300 mL) at room temperature, was treated with DeoxoFluor™ (10 µL, 0.055 mmol). The solution was stirred for 48h at rt. The solution was diluted with EtOAc, washed with water (2 × 10 mL) and saturated aqueous $NaHCO_3$ (1 × 10 mL), then dried over $MgSO_4$. Filtration of the mixture followed by evaporation of the solvent gave a dark orange oil which was purified by silica gel chromatography (pTLC, 500 µm, 1% triethylamine/50% hexane/49% EtOAc). The major UV active band was recovered to give the title compound as a yellow oil (2.9 mg, 34%). The reaction was monitored by HPLC-UV/MS using samples prepared from 745 µL of *i*PrOH and 5 µL of sample, then filtered through a syringe filter. **^{1}H NMR (800 MHz, CDCl3)** δ 7.44 – 7.36 (m, 4H), 6.94 (dd, 1H, 55 Hz $^2J_{HF}$), 4.61 (dd, J = 8.0, 6.0 Hz, 1H), 3.64 – 3.51 (m, 2H), 2.74 (s, 3H), 1.93-1.90 (m, 3H), 1.53 (s, 9H). **^{13}C NMR (201 MHz, CDCl3)** δ 129.6, 129.0, 110.2 (dd, 236 Hz $^1J_{FC}$), 77.2, 60.4, 54.0, 41.0, 37.7, 29.7, 28.2, 21.1, 14.8, 14.1, 11.9. HRMS (HESI-TOF) m/z calcd for $(M + H)^+$ 493.1277 , found 493.1268.

Preparation of *tert*-butyl (*S*)-2-(4-(4-chlorophenyl)-2-(fluoromethyl)-3,9-dimethyl-6*H*-thieno[3,2-*f*][1,2,4]triazolo[4,3-*a*][1,4]diazepin-6-yl)acetate (+)-JQ1-F$_1$ (7): A solution of JQ1-OH (8.3 mg, 0.017 mmol) in dichloromethane (DCM; 0.3 mL) was charged with DeoxoFluor (2 eq, 6.5 μL, 0.034 mmol) and stirred at room temperature for 24 hours. Conversion to the product was monitored via LC/MS. After the reaction reached to plateau, it was quenched saturated aqueous $NaHCO_3$ (2 mL) and extracted with DCM (3 × 5 mL). The combined DCM layer was dried over anhydrous sodium sulfate and then evaporated to provide 15 mg of crude material. The crude was subjected to preparative silica gel chromatography (pTLC) using 50% EtOAC and 50% Hexanes (with 0.1% triethylamine) to provide JQ1-F1 as a yellow oil (3.2 mg, 38% yield). **^{1}H NMR (800 MHz, CDCl3)** δ 7.40 (d, J = 8.2 Hz, 2H), 7.36 – 7.34 (m, 2H), 5.5 (d, 2H, 49 Hz $^2J_{HF}$), 4.58 (dd, J = 8.1, 6.0 Hz, 1H), 3.60 – 3.52 (m, 2H), 2.71 (s, 3H), 1.85 (d, J = 2.9 Hz, 3H), 1.50 (s, 9H). **^{13}C NMR (201 MHz, CDCl3)** δ 170.7, 163.2, 155.6, 149.9, 137.1, 129.7, 128.9, 81.1, 75.90 (d, 169 Hz $^1J_{FC}$), 54.0, 37.8, 29.7, 14.6, 11.9. HRMS (HESI-TOF) m/z calcd for $(M + H)^+$ 475.1371 , found 475.1374.

(+)-JQ1-D intermediates

Preparation of 2-amino-4-methyl-5-(trideuteriomethyl)-3-thienyl]-(4-chlorophenyl)methanone (10): A stirred solution of 3-(4-chlorophenyl)-3-oxo-propanenitrile (1.4 g, 7.79 mmol, 1.00 eq) in abs EtOH (15 mL) was treated with 4,4,4-trideuteriobutan-2-one (0.77 mL, 8.57 mmol, 1.10 eq), morpholine (0.67 mL, 7.79 mmol, 1.00 eq) and sulfur (249 mg, 7.79 mmol, 1.00 eq). The reaction mixture was stirred at 70 °C for 12 h. The mixture was cooled to rt and treated with brine (70 mL). The reaction mixture was extracted with EtOAc (3 × 50 mL). The combined organic phase was washed with brine (50 mL), dried over anhy. $MgSO_4$, filtered and concentrated under reduced pressure to a residue. The residue was chromatographed over silica gel (0% to 100% EtOAc/hex) to afford the title compound **10** (1.46 g, 70%) as a yellow solid. **^{1}H NMR (600 MHz, CDCl3)** δ 7.50 – 7.47 (m, 2H), 7.42 – 7.39 (m, 2H), 1.58 (s, 3H). ^{13}C NMR (151 MHz, CDCl3) δ 191.6, 163.0, 140.1, 136.7, 129.5, 117.2, 28.1, 15.5.

Preparation of tert-butyl (3S)-4-[[3-(4-chlorobenzoyl)-4-methyl-5-(trideuteriomethyl)-2-thienyl]amino]-3-(9H-fluoren-9-ylmethoxycarbonylamino)-4-oxo-butanoate (11): A solution of 2-amino-4-methyl-5-(trideuteriomethyl)-3-thienyl]-(4-chlorophenyl)methanone **10** (1.46 g, 5.43 mmol, 1.00 eq) in dry DCM (55 mL) was treated with (2*S*)-4-*tert*-butoxy-2-(9*H*-fluoren-9-ylmethoxycarbonylamino)-4-oxo-butanoic acid (2.24 g, 5.43 mmol, 1.00 eq). The reaction mixture was treated with EEDQ (2.03 g, 8.18 mmol, 1.50 eq). The reaction mixture was stirred at rt for 3d. A second charge of EEDQ (0.67 g, 2.72 mmol, 0.50 eq) was added and the reaction stirred at rt for another 1d to bring the total reaction time to 96 hours. Brine (100 mL) was added and the reaction mixture was extracted with DCM (3 × 50 mL). The combined organic layers were washed with brine (50 mL), dried over anhydrous $MgSO_4$, filtered and concentrated to a residue under reduced pressure. The product was taken to the next reaction without any purification.

Preparation of tert-butyl (3S)-3-amino-4-[[3-(4-chlorobenzoyl)-4-methyl-5-(trideuteriomethyl)-2-thienyl]amino]-4-oxo-butanoate (12): Compound **11** (2.67g, 4.04 mmol, 1.00 eq) was dissolved in a solution of 20% piperidine in DMF (27 mL) and was stirred for 30 minutes at 23 ºC. After 30 minutes, ethyl acetate (35 mL) and brine (35 mL) were added to the reaction mixture. The aqueous layer was washed with ethyl acetate (2 × 50 mL). The combined organic layers were washed with brine (50 mL), then dried over anhydrous magnesium sulfate, filtered and concentrated under reduced pressure. The product was purified by flash column chromatography to afford the compound **12** (1.160 g, 65%) as a brown oil. **^{1}H NMR (600 MHz, CDCl3)** δ 7.62 – 7.59 (m, 2H), 7.46 – 7.43 (m, 2H), 3.85 (dd, J = 7.7, 3.7 Hz, 1H), 2.90 (dd, J = 16.7, 3.7 Hz, 1H), 2.75 (dd, J = 16.7, 7.8 Hz, 1H), 1.74 (s, 3H), 1.45 (s, 9H).

Preparation of tert-butyl2-[(3S)-5-(4-chlorophenyl)-6-methyl-2-oxo-7-(trideuteriomethyl)-1,3-dihydrothieno[2,3-e][1,4]diazepin-3-yl]acetate (13): Compound **12** (1.160 g, 2.64 mmol, 1.00 eq) was dissolved in toluene (33 mL). Silica gel (1.50 g) was added to the reaction mixture and was stirred for 3h at 90 ºC. After 3h, the reaction was cooled to rt. The silica gel was removed using solid-phase extraction (SPE) and washed with

EtOAc. The combined filtrates were concentrated to afford compound **13** (1.08 g, 97%) as a brown oil. **^{1}H NMR (600 MHz, CDCl3)** δ 7.43-7.41 (m, 2H), 7.35 – 7.31 (m, 2H), 4.24 – 4.19 (m, 1H), 3.40-3.34 (m, 1H), 3.13-3.08 (m, 1H), 1.60 (s, 3H), 1.48 (s, 9H). **^{13}C NMR (151 MHz, CDCl3)** δ 171.1, 169.2, 165.0, 141.0, 136.9, 136.4, 130.2, 129.7, 128.6, 127.7, 126.4, 80.7, 61.2, 37.5, 28.2, 14.4.

Preparation of tert-butyl 2-[(9S)-7-(4-chlorophenyl)-5,13-dimethyl-4-(trideuteriomethyl)-3-thia-1,8,11,12-tetrazatricyclo[8.3.0.02,6]trideca-2(6),4,7,10,12-pentaen-9-yl]acetate, (+) JQ1-D (14): Compound **13** (1.08 g, 2.56 mmol, 1.00 eq) was added to a −78 ºC solution of *tert*-butoxypotassium (431 mg, 3.84 mmol, 1.50 eq) in THF (25 mL). The reaction mixture was warmed to −10 ºC, and then stirred at 23 ºC for 45 minutes. The reaction was cooled to −78 ºC and treated with diethyl chlorophosphate (0.557 mL, 3.84 mmol, 1.50 eq). The reaction was warmed to −10 ºC over 1 h and then acetic hydrazide (284 mg, 3.84 mmol, 1.50 eq) was added to the stirring solution and stirred at rt for an additional 1 h. 1-butanol (10 mL) was added to the reaction mixture and stirred for 90 min. All solvents were removed under reduced pressure. The product was purified by flash column chromatography (0 to 100% EtOAc/hex) to afford a nearly enantiomerically pure **(+)-JQ1-D**. **^{1}H NMR (800 MHz, CDCl3)** δ 7.41 (d, J = 8.1 Hz, 2H), 7.35 – 7.31 (m, 2H), 4.56 (dd, J = 7.9, 6.1 Hz, 1H), 3.58 – 3.52 (m, 2H), 2.67 (s, 3H), 1.69 (s, 3H), 1.50 (s, 9H). **^{13}C NMR (201 MHz, CDCl3)** δ 170.9, 163.6, 155.5, 149.8, 136.7, 132.4, 130.9, 130.5, 130.4, 129.8, 128.7, 116.7, 80.9, 60.4, 53.9, 37.9, 28.2, 14.4, 11.9. HRMS (HESI-TOF) m/z calcd for $(M + H)^{+}$ 460.1653 , found 460.1645.

ALPHA Assay Conditions

The ALPHA Assay was performed as published by Roberts and Bradner[34] with a 40 nM protein concentration, 20 nM Biotin-JQ1 concentration and with nickel acceptor beads and streptavidin donor beads at 25 µg/ml each.

Metabolic stability of (+)-JQ1 derivatives in liver microsomes.

(+)-JQ1 and modified compounds (0.2 μM) were incubated in the mouse or human liver microsomes (0.1 mg protein/mL) at 37 °C. Given the slower rate of metabolism for JQ1-D, we used 0.5 mg protein/ mL. The samples are collected at specific time-points (0, 5, 10, 20, 40, and 60 min. The reactions are terminated by adding equivalent volume of ice-cold MeOH and vortexed. The reaction mixtures are centrifuged at rcf 15,000 for 15 min. Five μL of the supernatant will be analyzed by UHPLC-Q Exactive Orbitrap MS (Thermo Fisher Scientific, San Jose, CA) equipped with 50 mm × 4.6 mm column (XDB C-18, Agilent Technologies, Santa Clara, CA). The column temperature is maintained at 40 °C. The flow rate of is at 0.3 mL/min with a 30% mobile phase (acetonitrile containing 0.1% formic acid). Q Exactive MS is operated in positive or negative mode with electrospray ionization. Ultrapure nitrogen is applied as the sheath (45 arbitrary unit), auxiliary (10 arbitrary unit), sweep (1.0 arbitrary unit) and the collision gas. The capillary gas temperature is set at 275 °C and the capillary voltage was set at 3.7 kV. MS data were acquired from 80 to 1200 Da in profile mode.

SPR binding analysis of (+) JQ1-F analogs

The Surface Plasmon Resonance (SPR) experiment was conducted using a Pioneer FE SPR System (Sartorius) equipped with a SPR HisCap Sensor Chip. The instrument was calibrated and validated prior to the experiment according to the manufacturer's instructions. The chip surface was activated with a mixture of N-hydroxysuccinimide (NHS), 50 mM, and N-ethyl-N'-(3-dimethylaminopropyl) carbodiimide (EDC), 200 mM, at a 20 □L/min flow rate for 5. minutes. Covalent attachment of his-BRDT to channel 1 was achieved by injecting 150 □L of protein at 10 mg/mL at 10 mL/min. Channel 2 of the chip was used as a blank reference and unreacted functional groups of both channels were blocked using ethanolamine. SPR running buffer consisted of 100 mM Tris-HCl, 200 mM NaCl, and 0.05% Tween-20. The JQ1 analog samples were prepared in a solution that closely mimics the buffer conditions and injected at 40□L/min using the OneStep injection system at 0.5, 5, and 50 □M concentrations. The real-

time binding response was recorded using the instrument software and sensorgrams were analyzed using the QDAT software. The baseline subtraction, referencing, and double-referencing techniques were applied to obtain specific binding responses. Kinetic parameters were calculated by fitting sensograms to OneStep binding models for determining the dissociation rate constant (k_d) for the highest concentration and constraining the lower concentrations to the determined value. All data was locally fitted to a simple 1:1 interaction model.

Pharmacokinetic studies in mice

Mouse studies were performed under protocols approved by the Institutional Animal Care and Use Committee of Baylor College of Medicine and guidelines established by the NIH Guide for the Care and Use of Laboratory Animals. Animals were housed under standard conditions of a 12-hour light/dark cycle in a vivarium with controlled ambient temperature (70˚ ± 2˚ and 20-70% relative humidity). Pharmacokinetic studies were performed using 8-12-week-old mice (C57BL/6NJ, female, n =4 and male n = 4)). Mice were administered 50 mg/kg of compounds (1:1 of mixtures of (+)-JQ1 and (+)-JQ1-D) via intraperitoneal injection to mice. Twenty µl of blood was collected via the tail vein at 0, 15, 30 minutes, 1, 2, 4, 8, and 24 hours post treatment. To 10 ul of plasma was added 25 ul of MeOH with internal standard. The resulting mixtures were vortexed and span at rcf 15,000 for 15 min and 5 ul of the supernatant were injected into the UHPLC-Q Exactive Orbitrap MS for analysis. The concentrations of tested compounds were calculated based on their corresponding standard curves. Pharmacokinetic parameters including half-time ($t_{1/2}$), area under the plasma concentration–time curve during the period of observation (AUC0–t), clearance normalized by bioavailability (CL/F) were calculated on WinNonlin software (Certara, Princeton, NJ) by noncompartmental analysis.

NMR Data

Figure S1. (+)-JQ1-CHO (**4**) 1H NMR Spectrum.

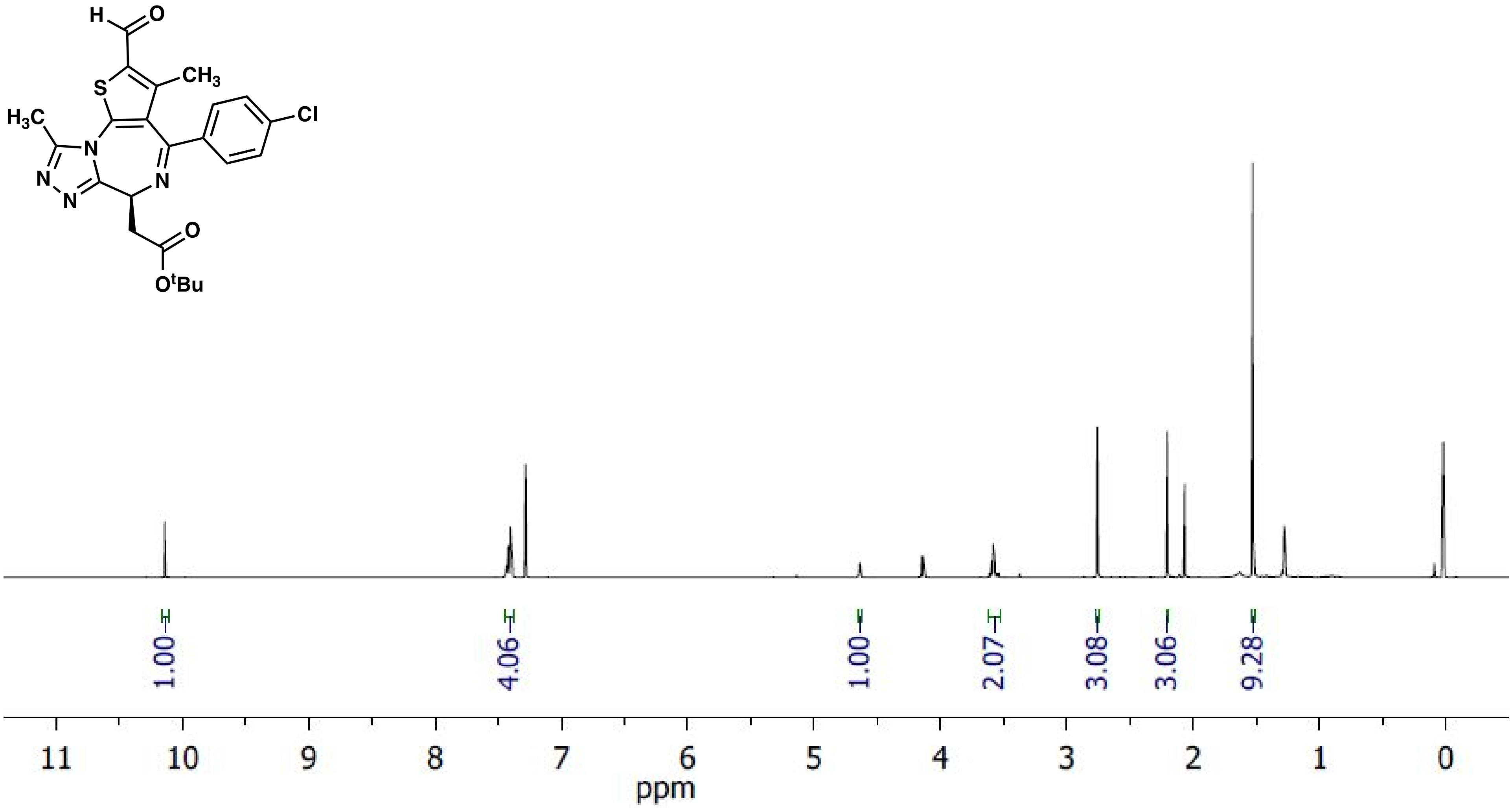

Figure S2. (+)-JQ1-CHO (**4**) 13C DEPT 135 NMR Spectrum

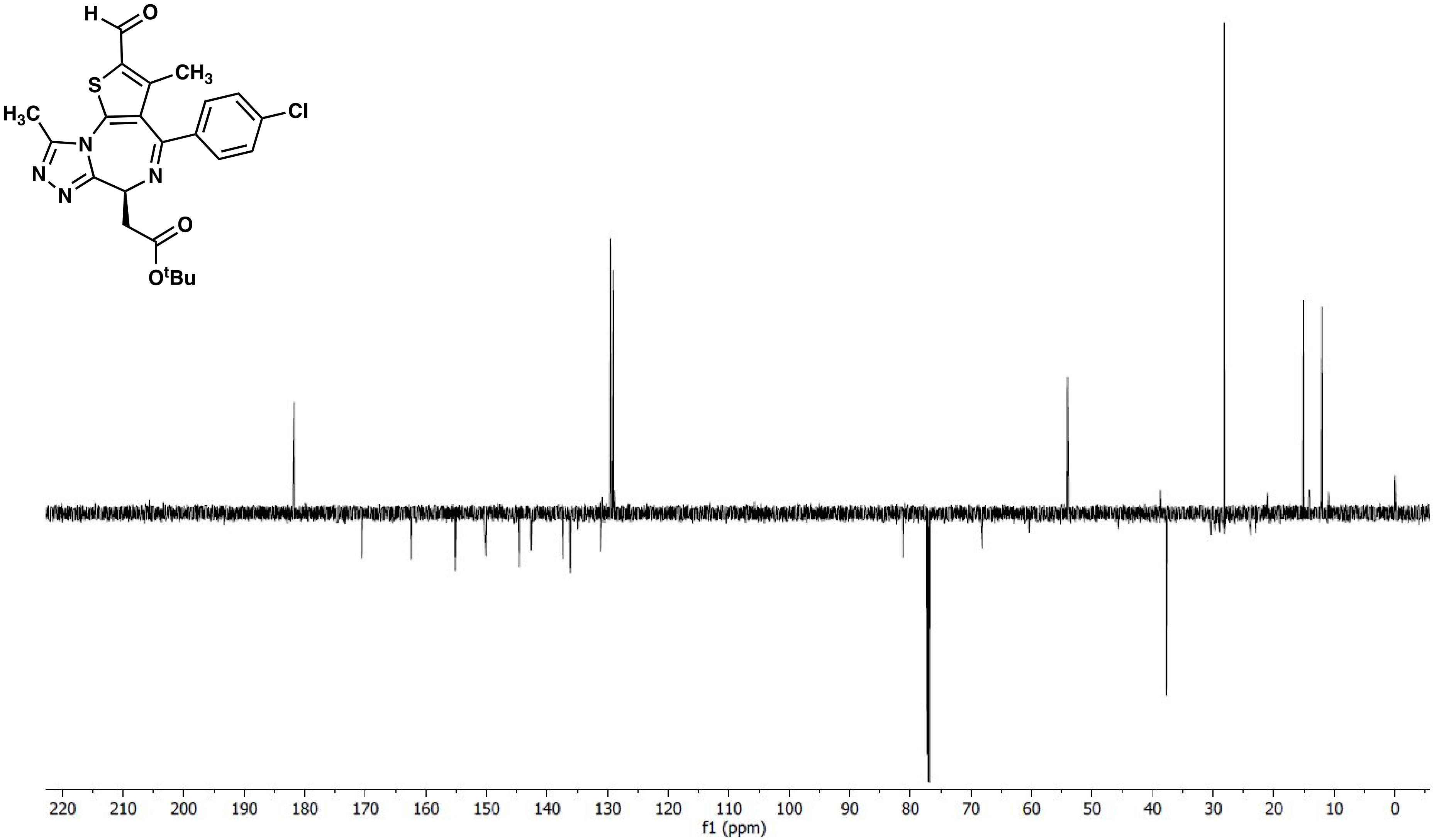

Figure S3. (+)-JQ1-CHO (**4**) HMBC NMR Spectrum

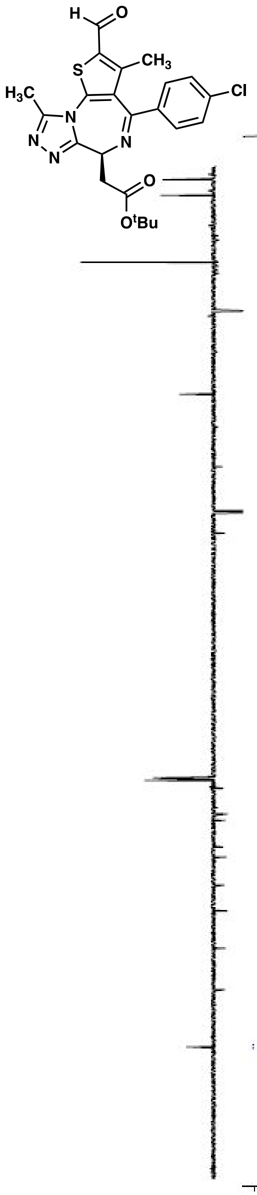

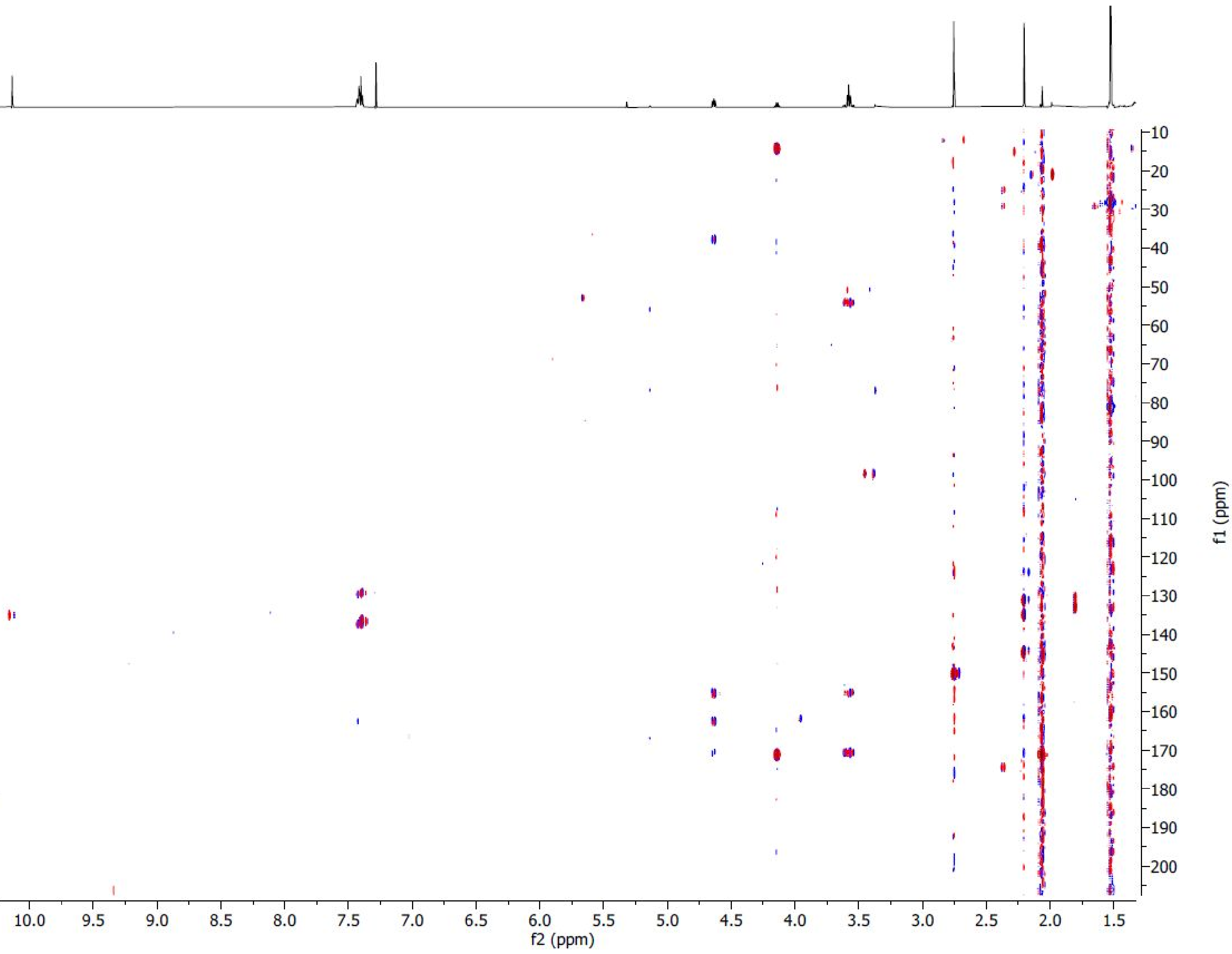

Figure S4. (+)-JQ1-CHO (**4**) HSQC NMR Spectrum

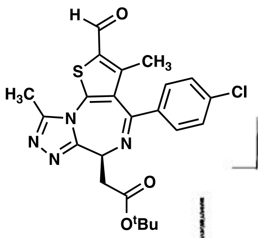

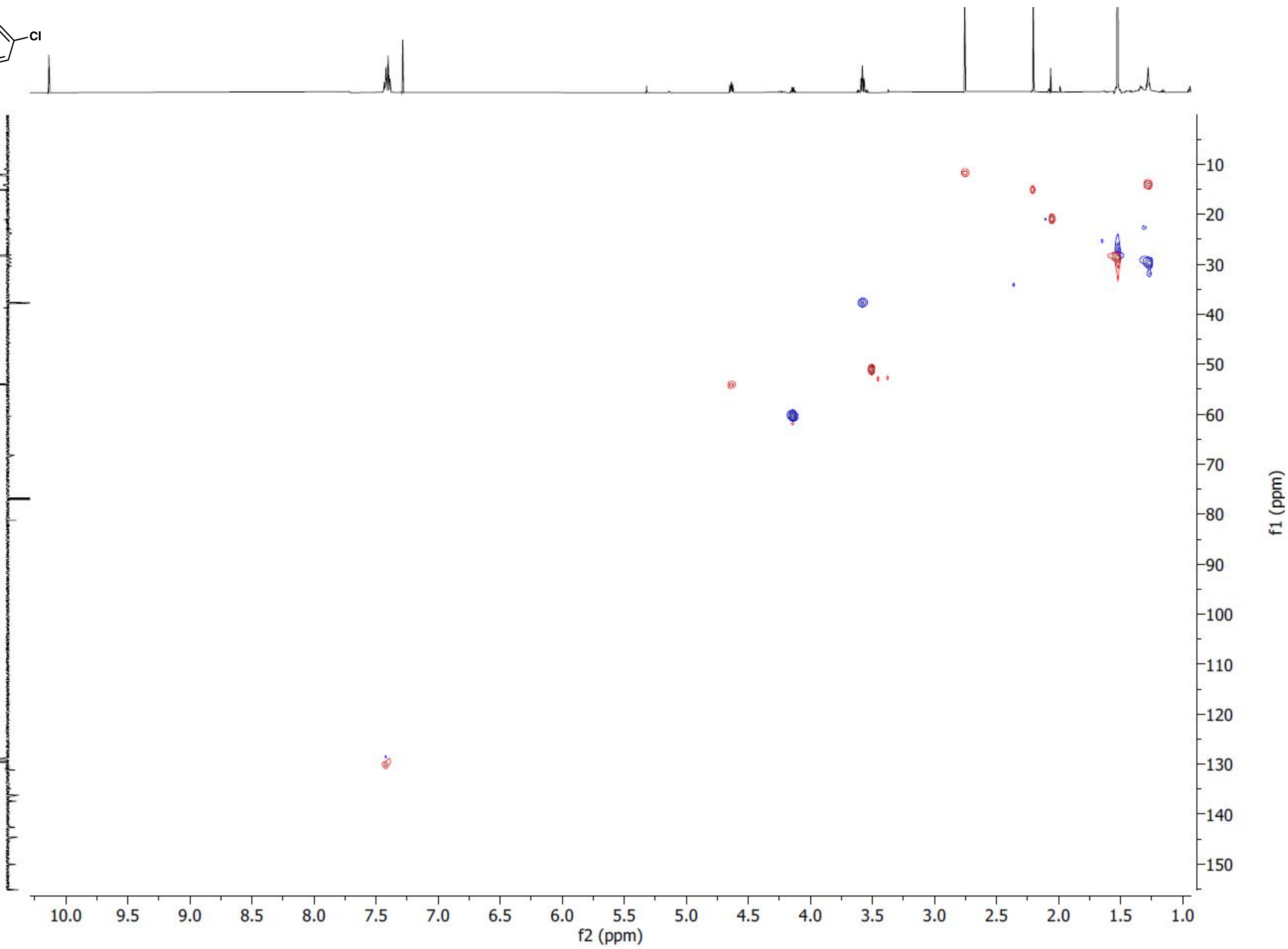

Figure S5. (+)-JQ1-CHO (**4**) NMR NOESY Spectrum

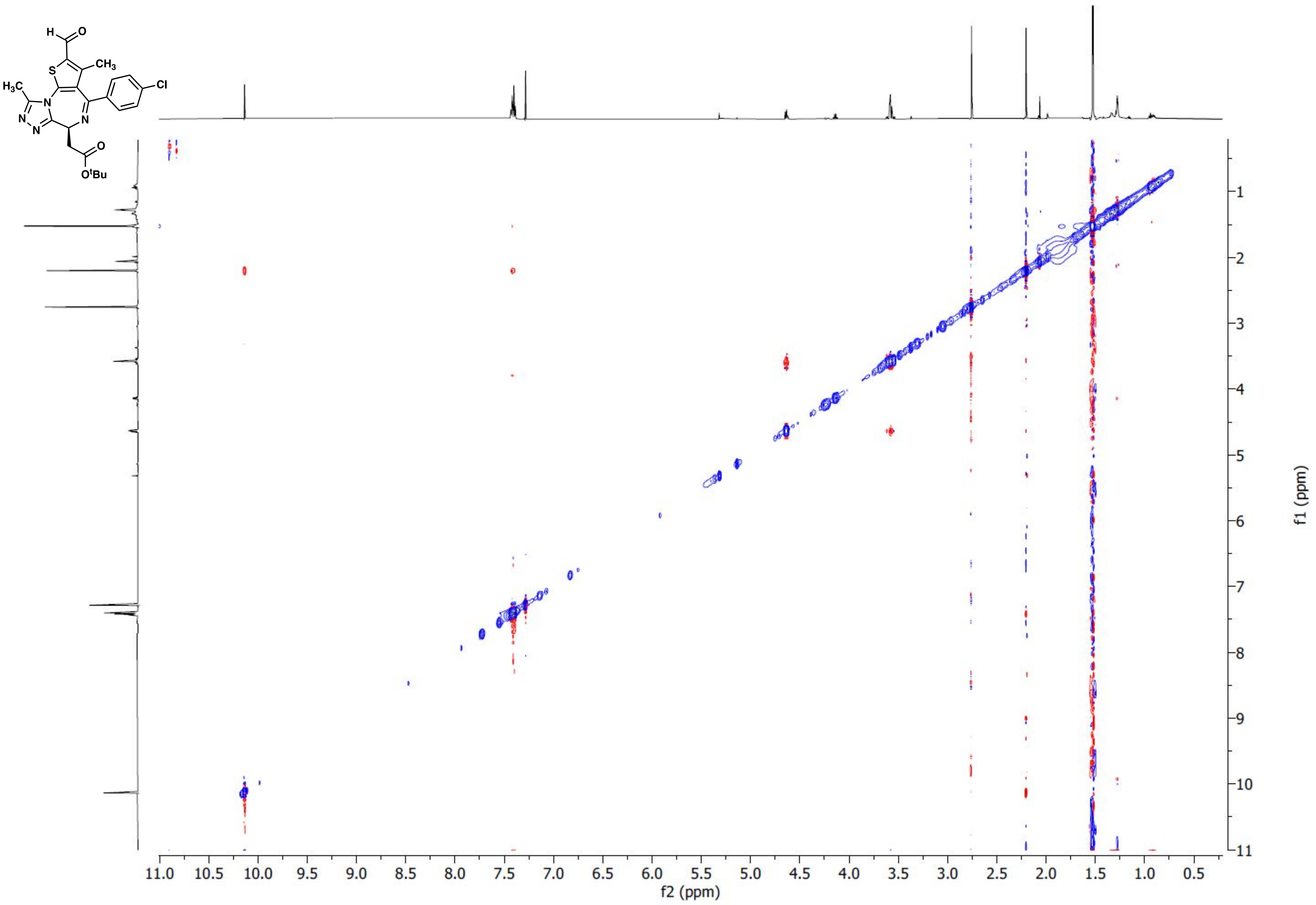

Figure S6. (+)-JQ1-OH **(5)** 1H NMR Spectrum.

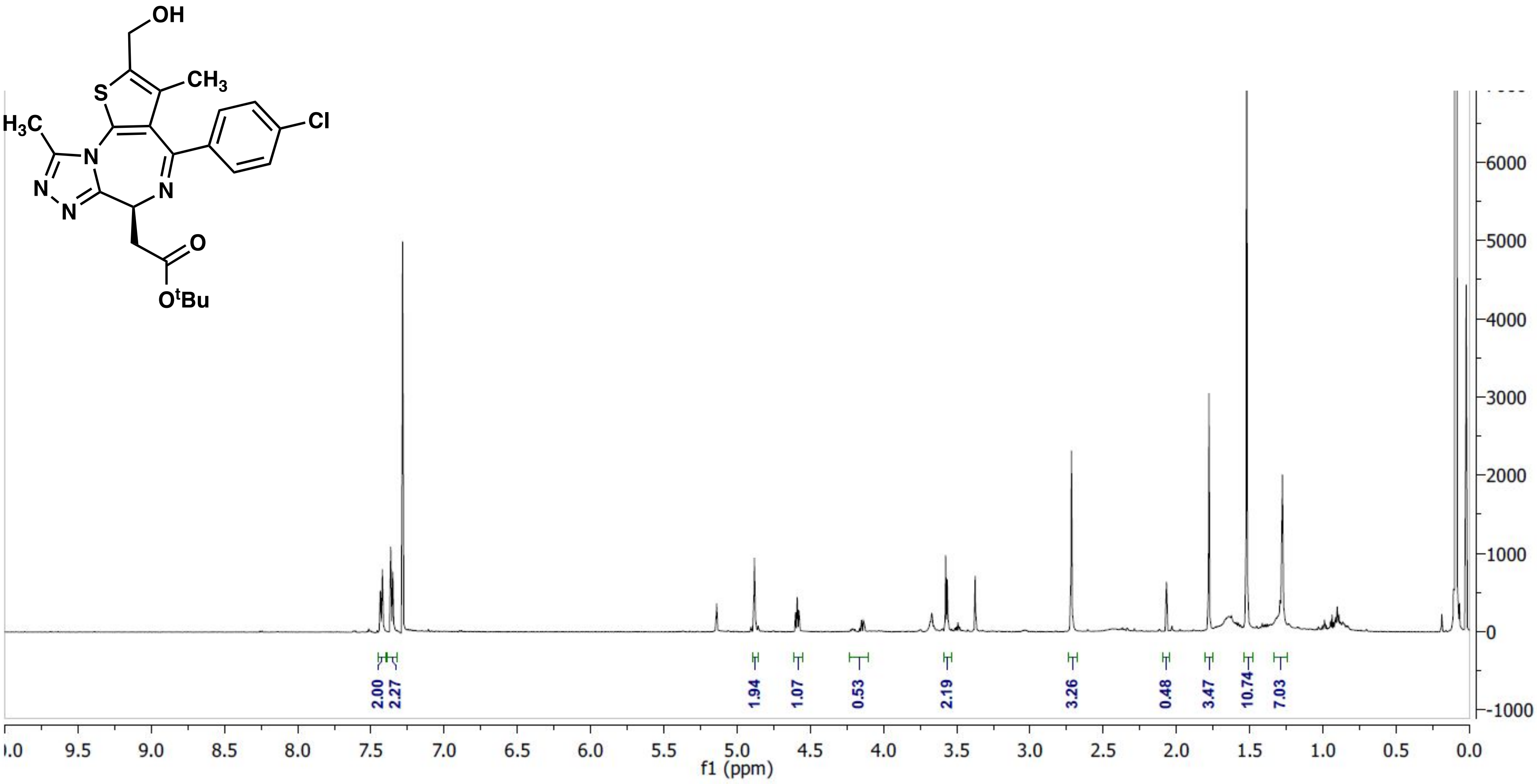

Figure S7. (+)-JQ1-F_2 **(6)** 1H NMR Spectrum

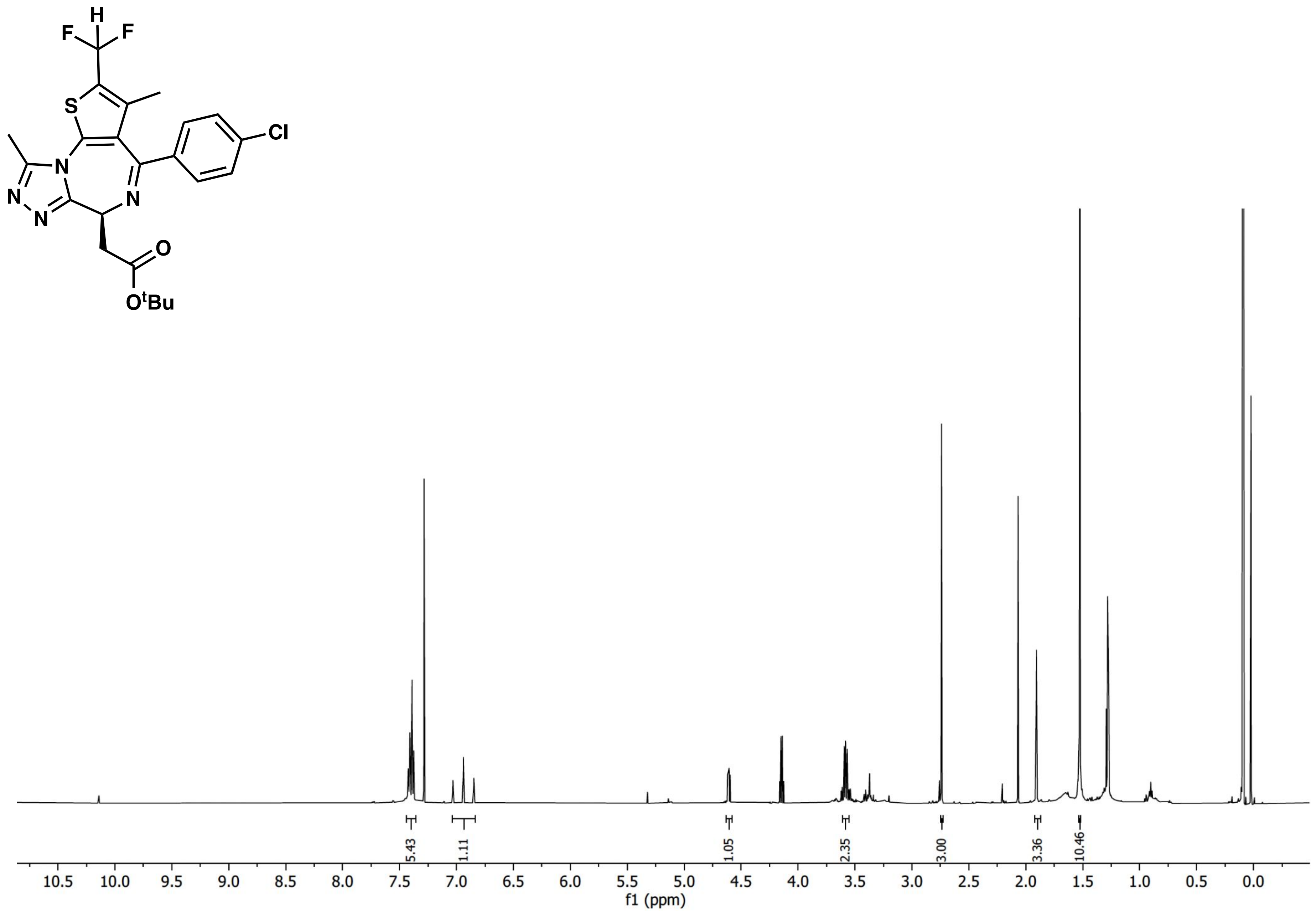

Figure S8. (+)-JQ1-F_2 **(6)** 13C DEPT 13 NMR Spectrum

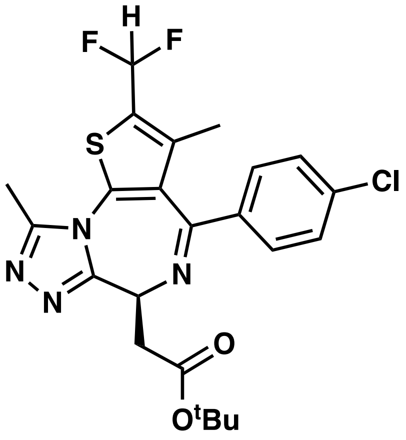

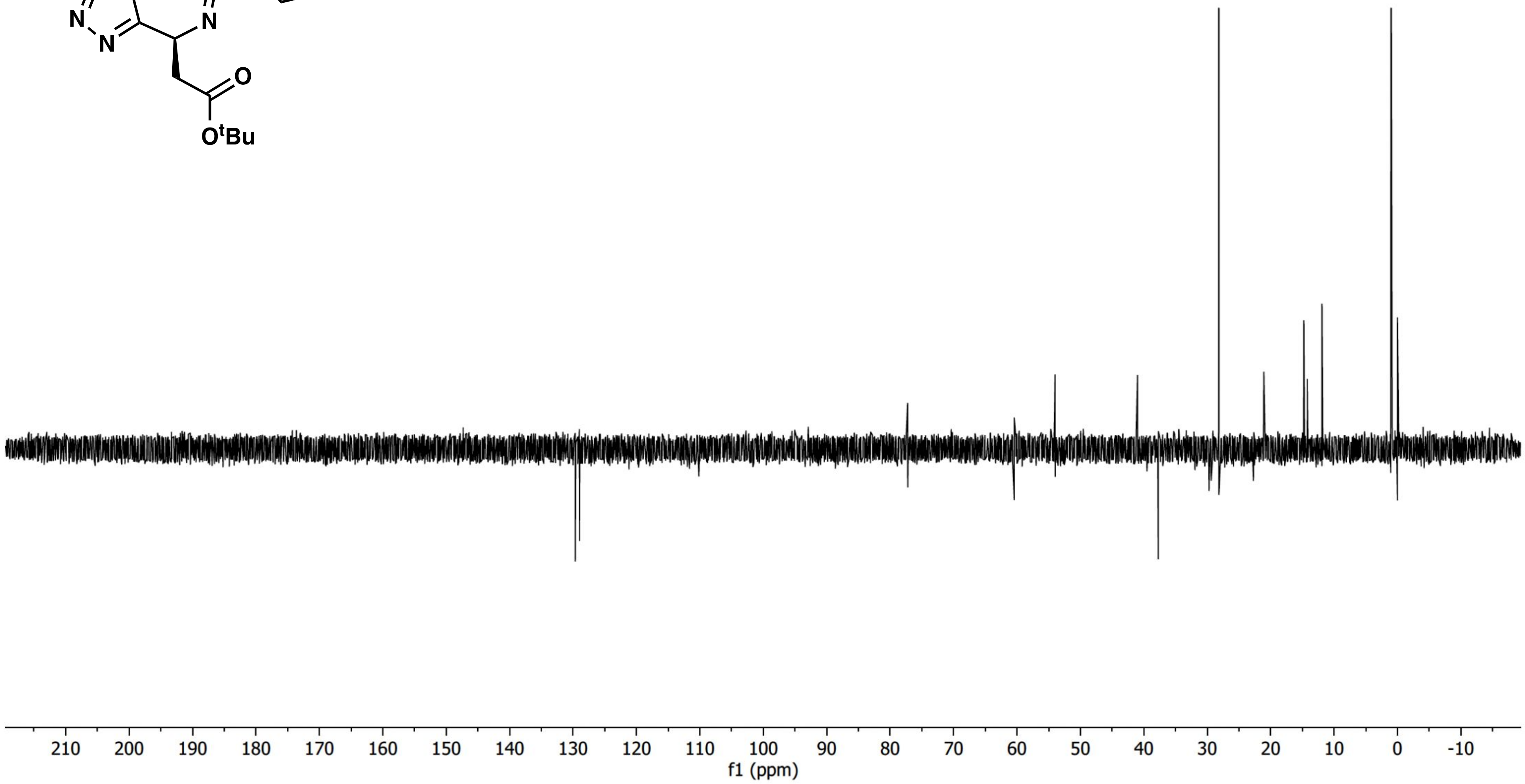

Figure S9. (+)-JQ1-F_2 **(6)** HSQC DEPT 13 NMR Spectrum

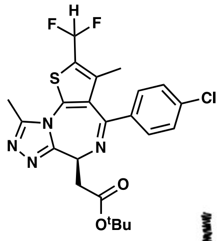

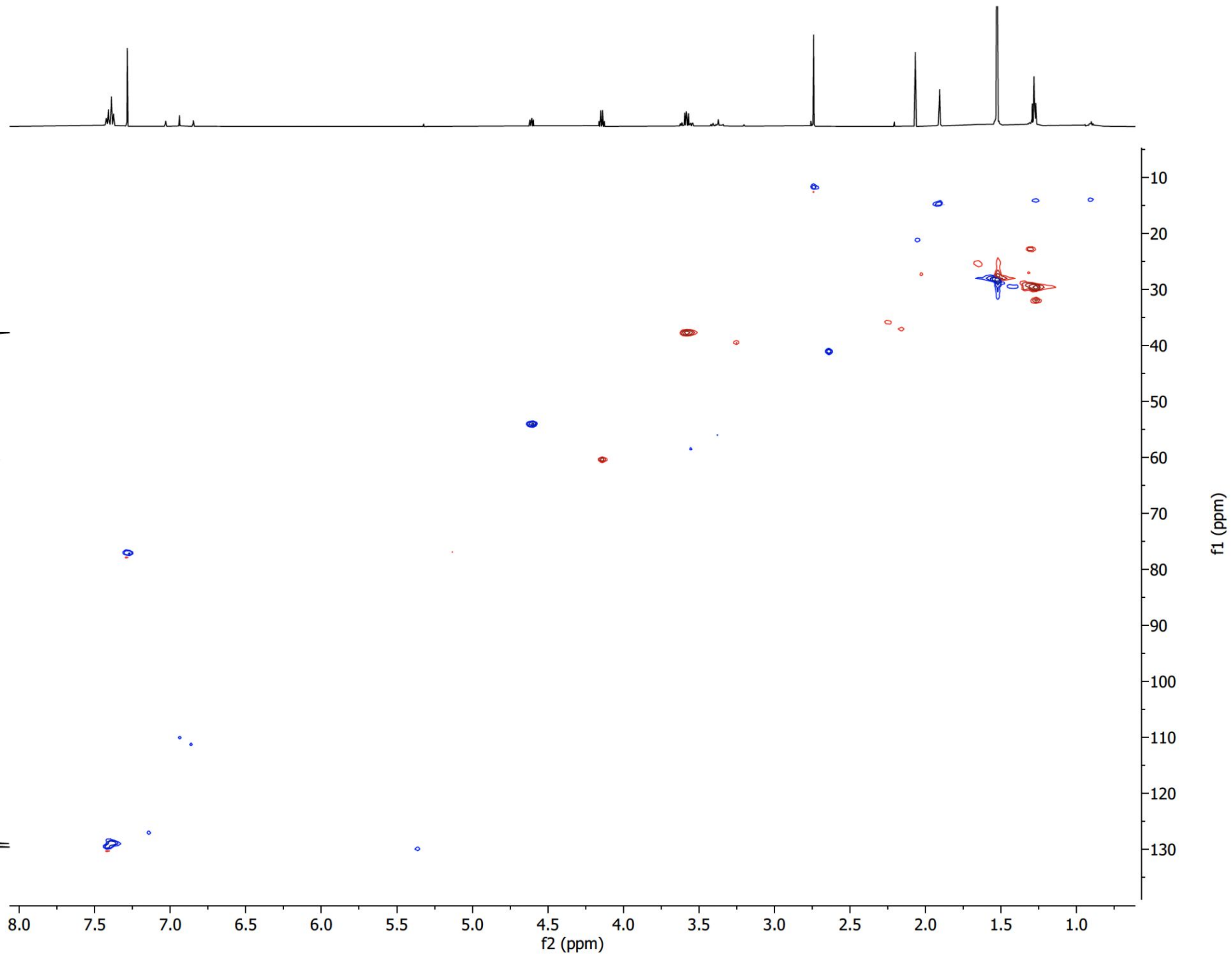

Figure S10. (+)-JQ1-F_1 **(7)** 1H NMR Spectrum

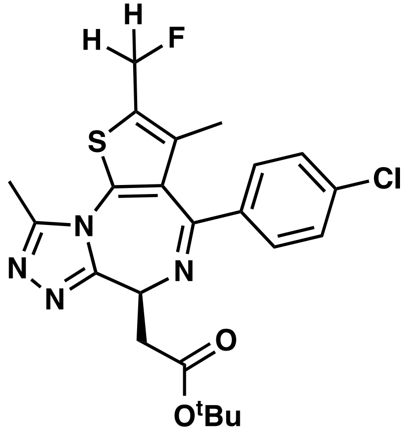

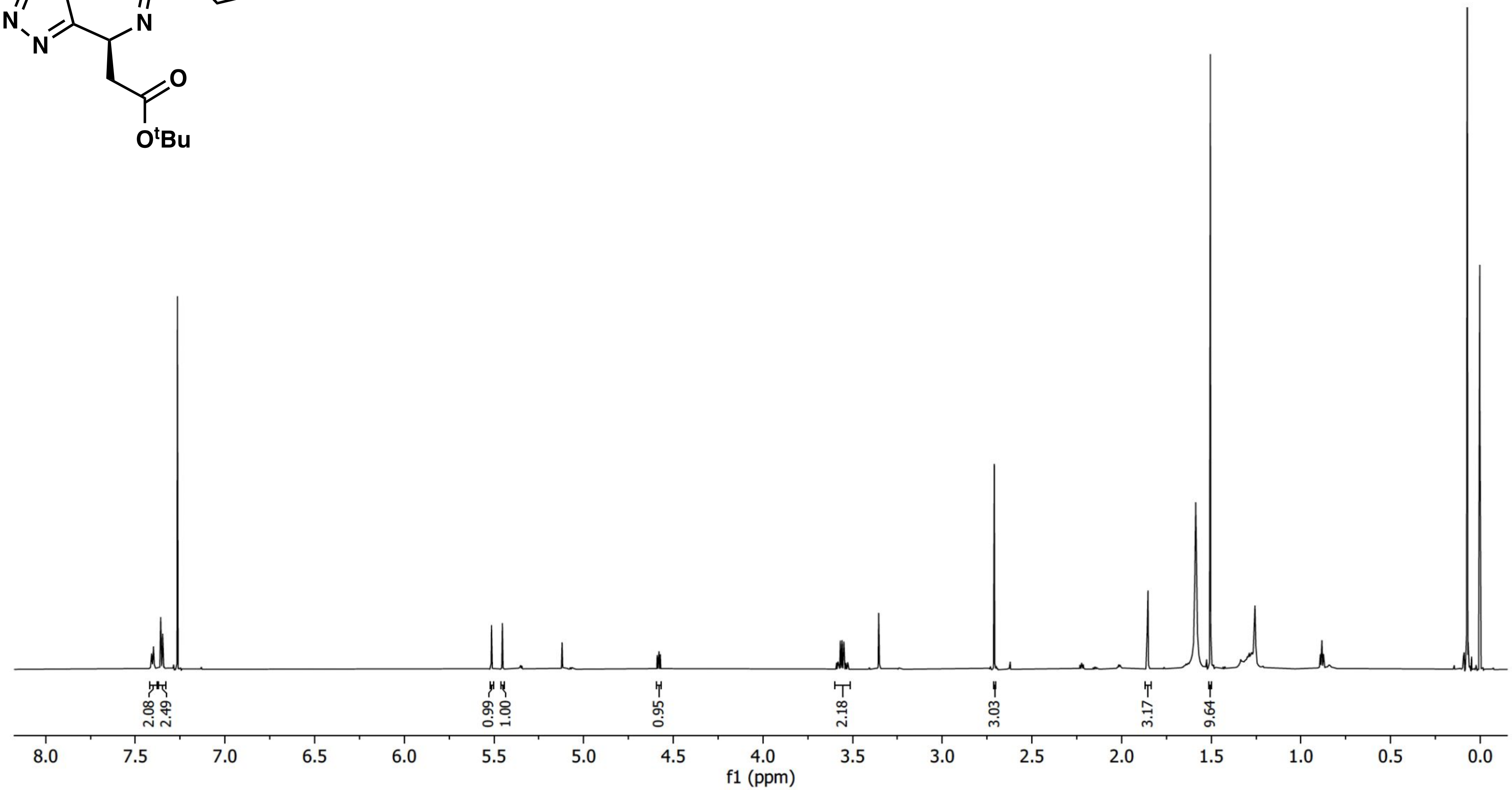

Figure S11. (+)-JQ1-F_1 **(7)** 13C DEPT 135 NMR Spectra

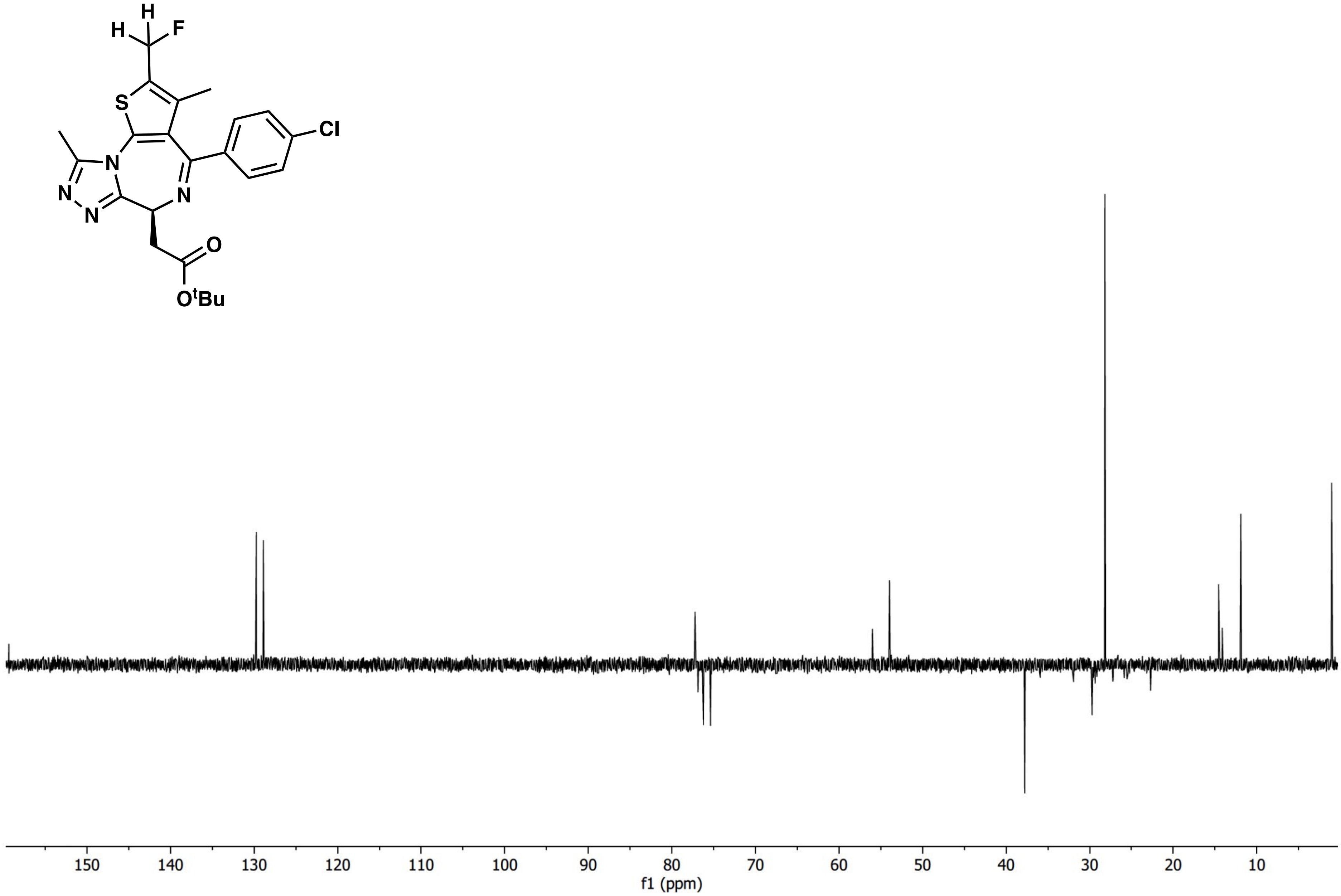

Figure S12. (+)-JQ1-F_1 **(7)** HSQC NMR Spectrum.

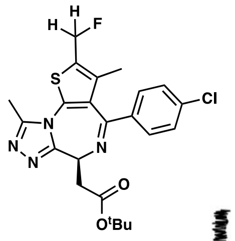

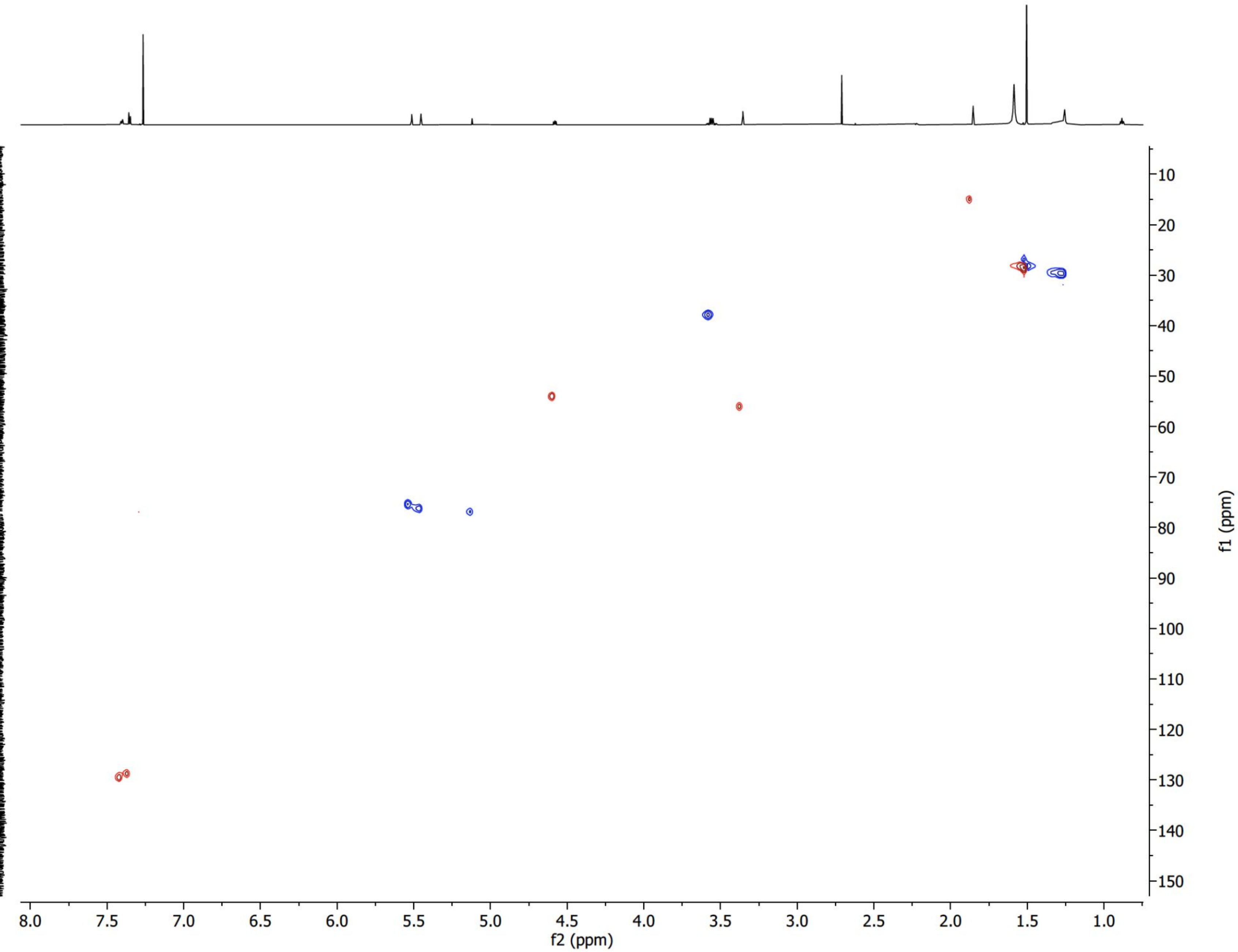

Figure S13. SPR binding data for (+)-JQ1-F analogs

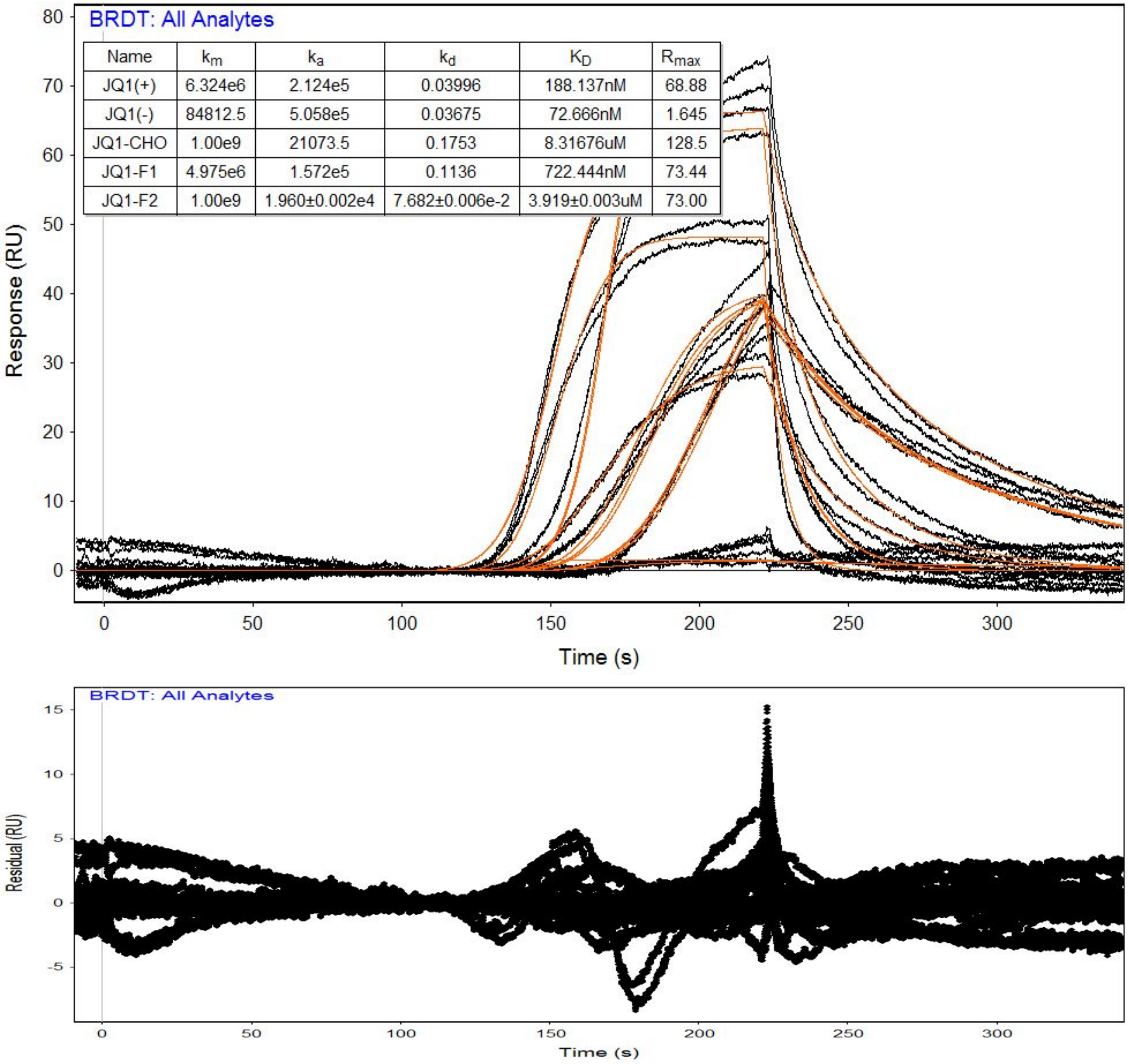

Figure S14. 2-amino-4-methyl-5-(trideuteriomethyl)-3-thienyl]-(4-chlorophenyl)methanone (**10**) 1H NMR spectrum

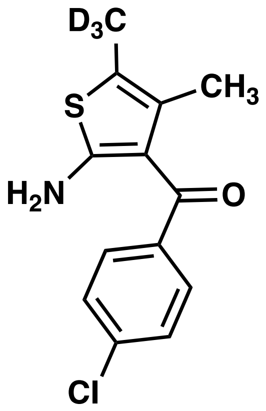

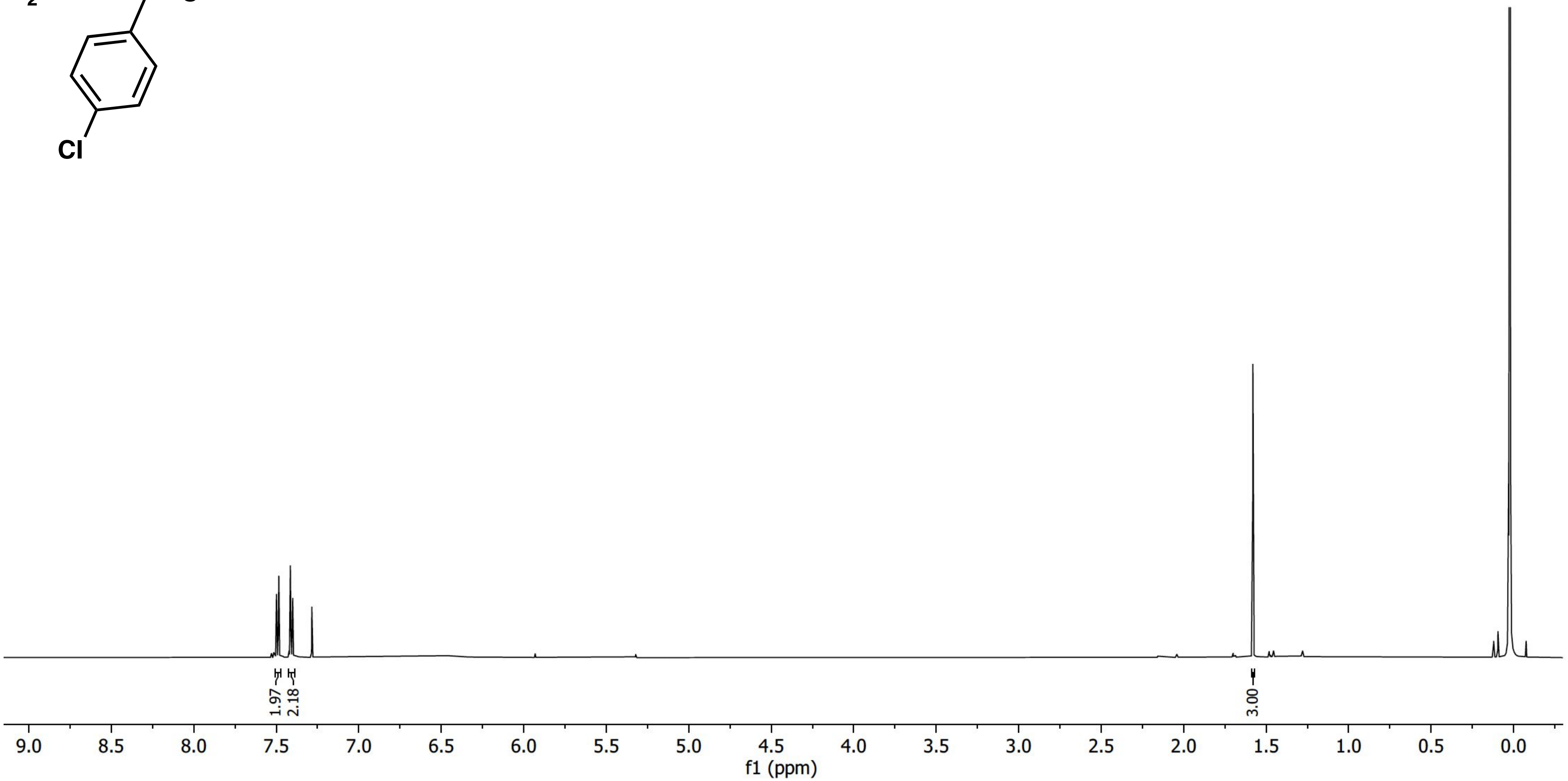

Figure S15. 2-amino-4-methyl-5-(trideuteriomethyl)-3-thienyl]-(4-chlorophenyl)methanone (**10**) HMBC NMR

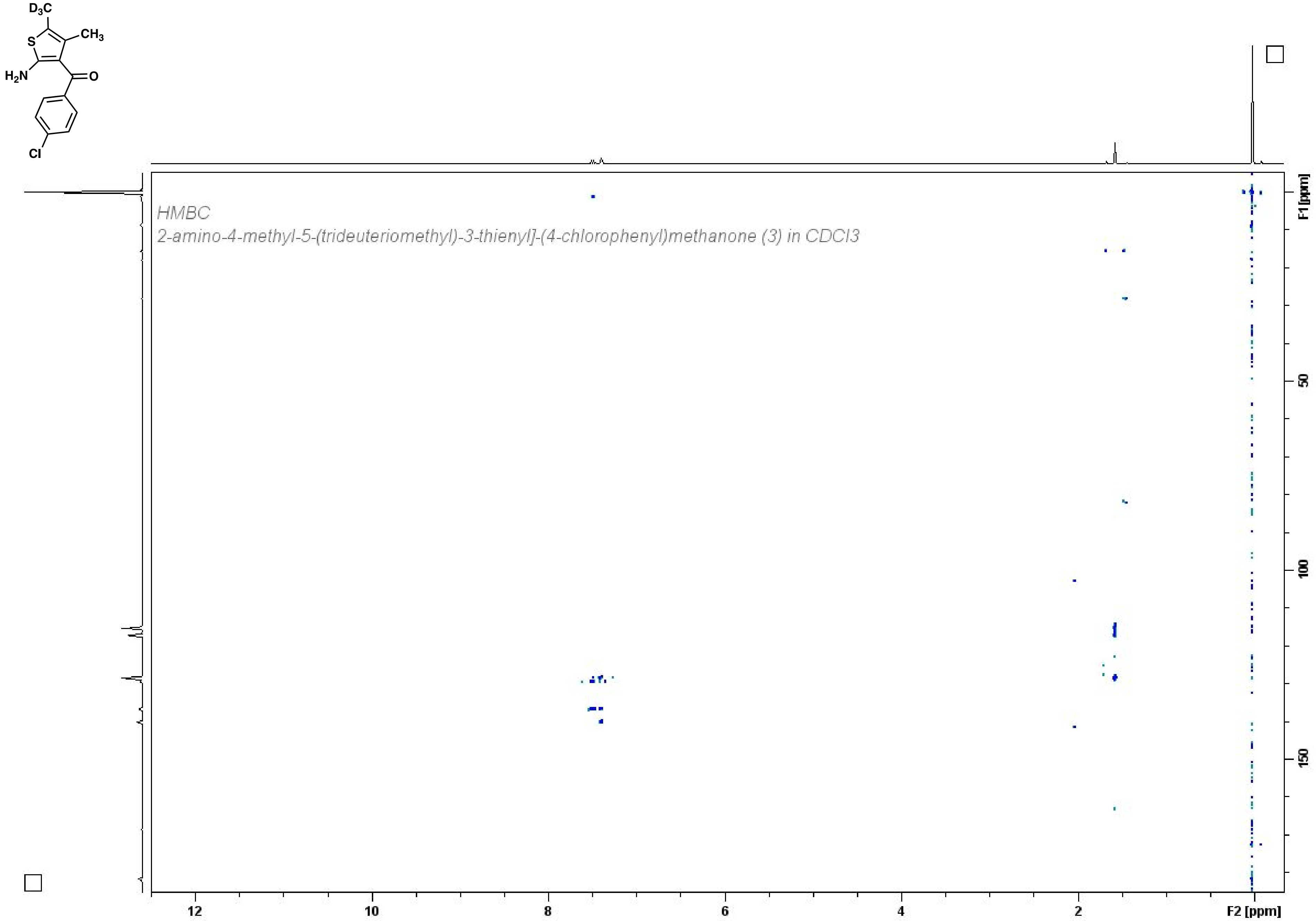

Figure S16. 2-amino-4-methyl-5-(trideuteriomethyl)-3-thienyl]-(4-chlorophenyl)methanone (**10**) HSQC NMR spectrum

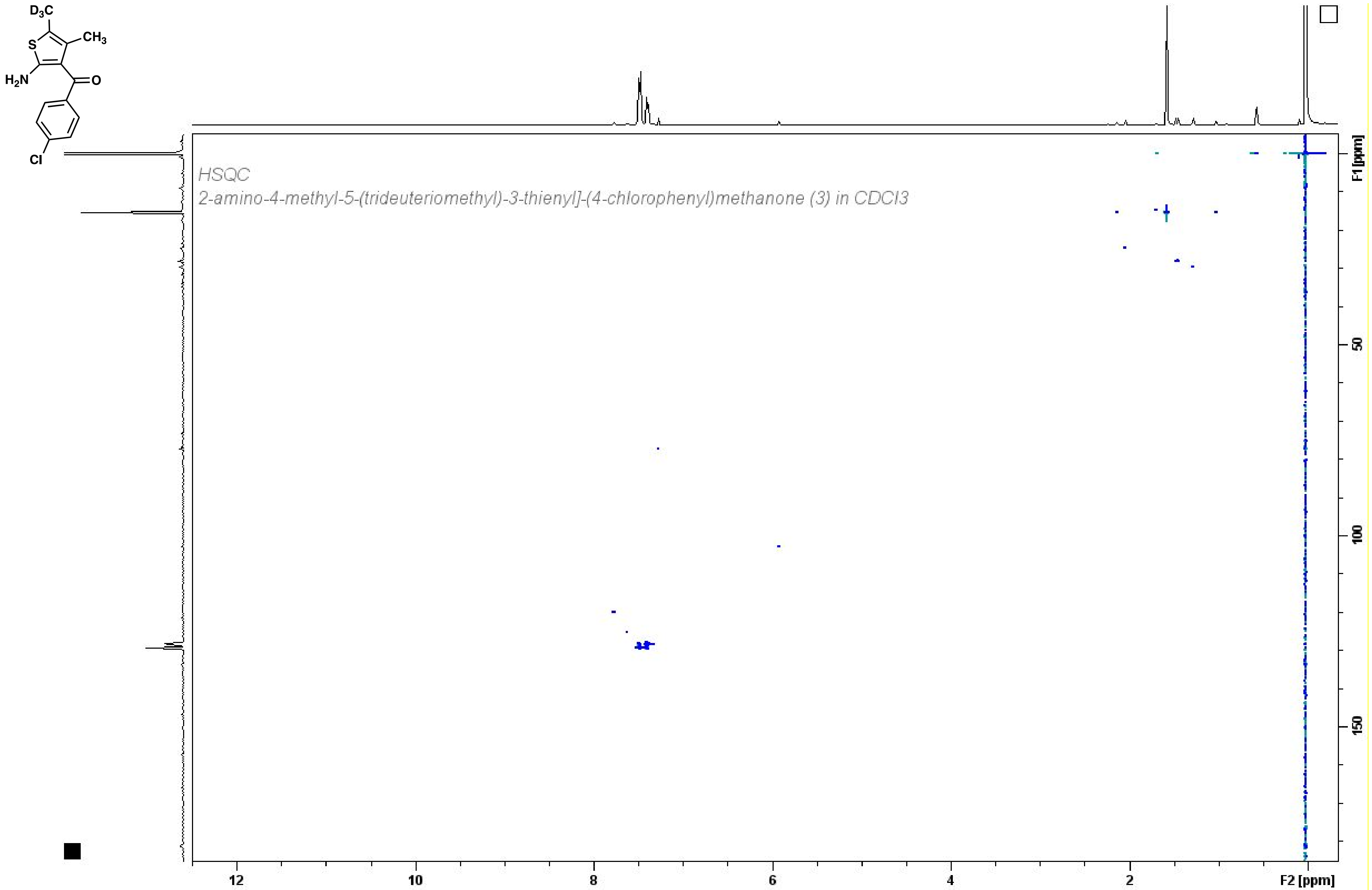

Figure S17. 2-amino-4-methyl-5-(trideuteriomethyl)-3-thienyl]-(4-chlorophenyl)methanone (**10**) NOESY NMR spectrum

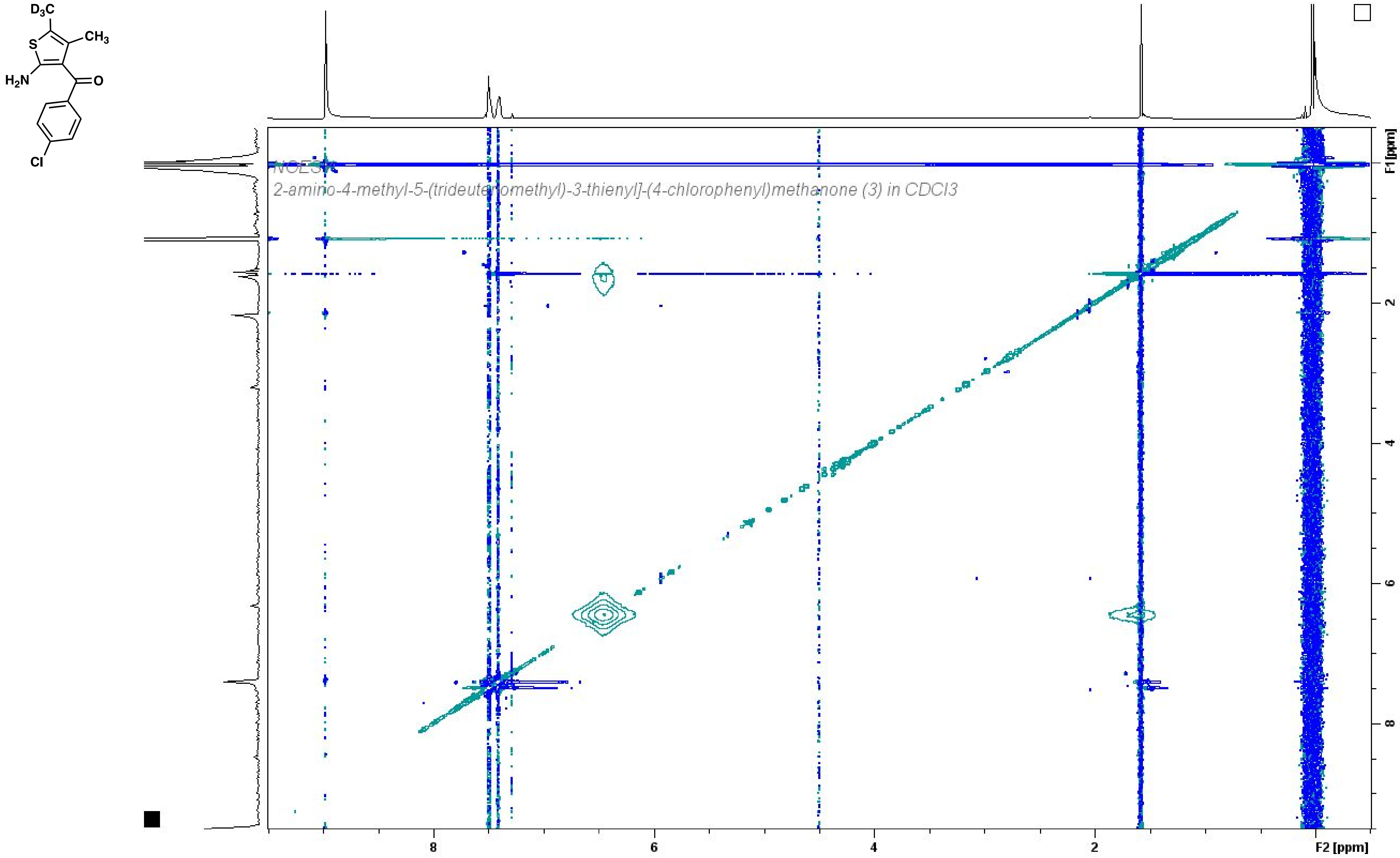

Figure S18. tert-butyl (3S)-3-amino-4-[[3-(4-chlorobenzoyl)-4-methyl-5-(trideuteriomethyl)-2-thienyl]amino]-4-oxo-butanoate (**12**) 1H NMR spectrum

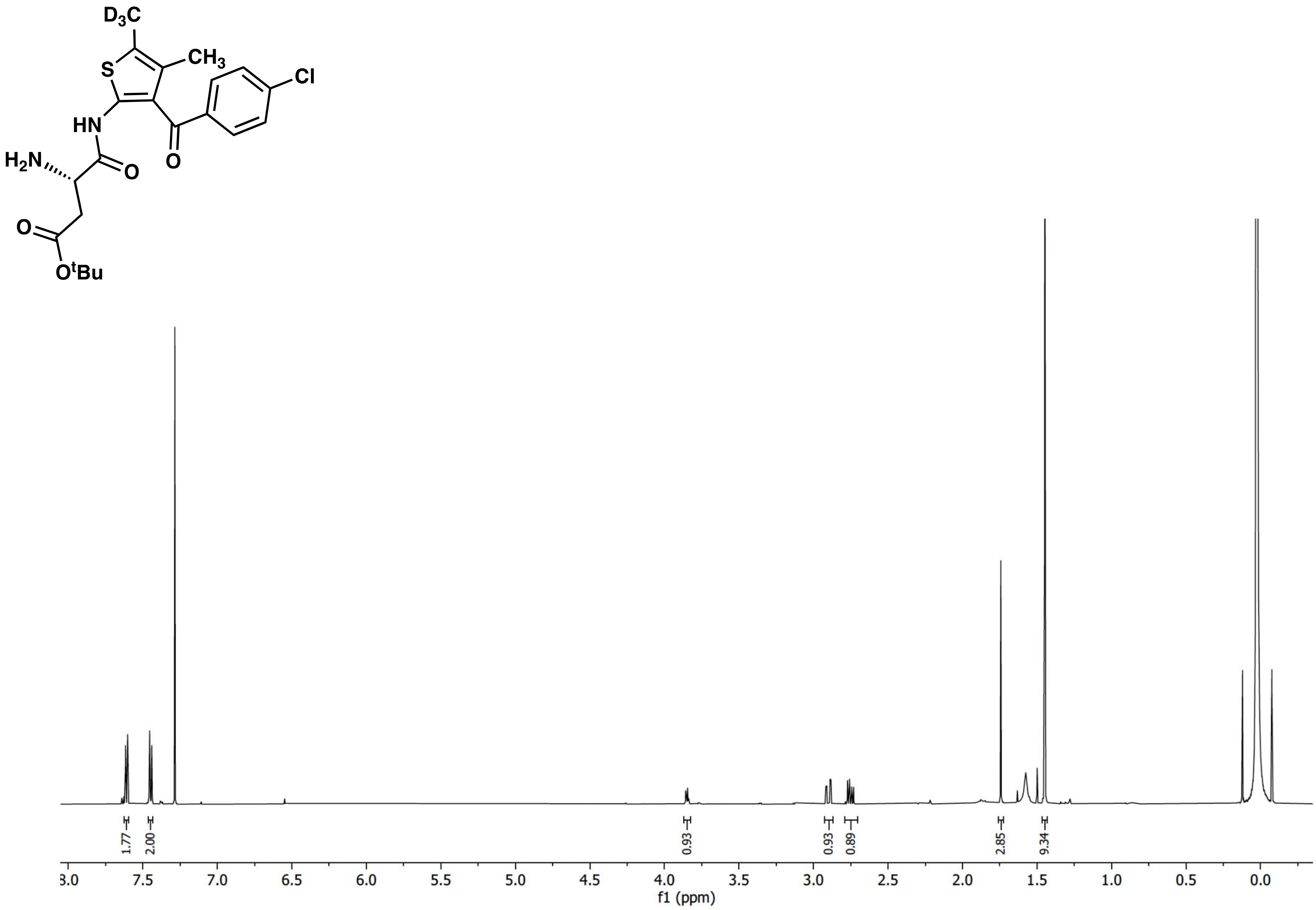

Figure S19. tert-butyl (3S)-3-amino-4-[[3-(4-chlorobenzoyl)-4-methyl-5-(trideuteriomethyl)-2-thienyl]amino]-4-oxo-butanoate (**12**) 13C DEPTQ-135 NMR spectrum

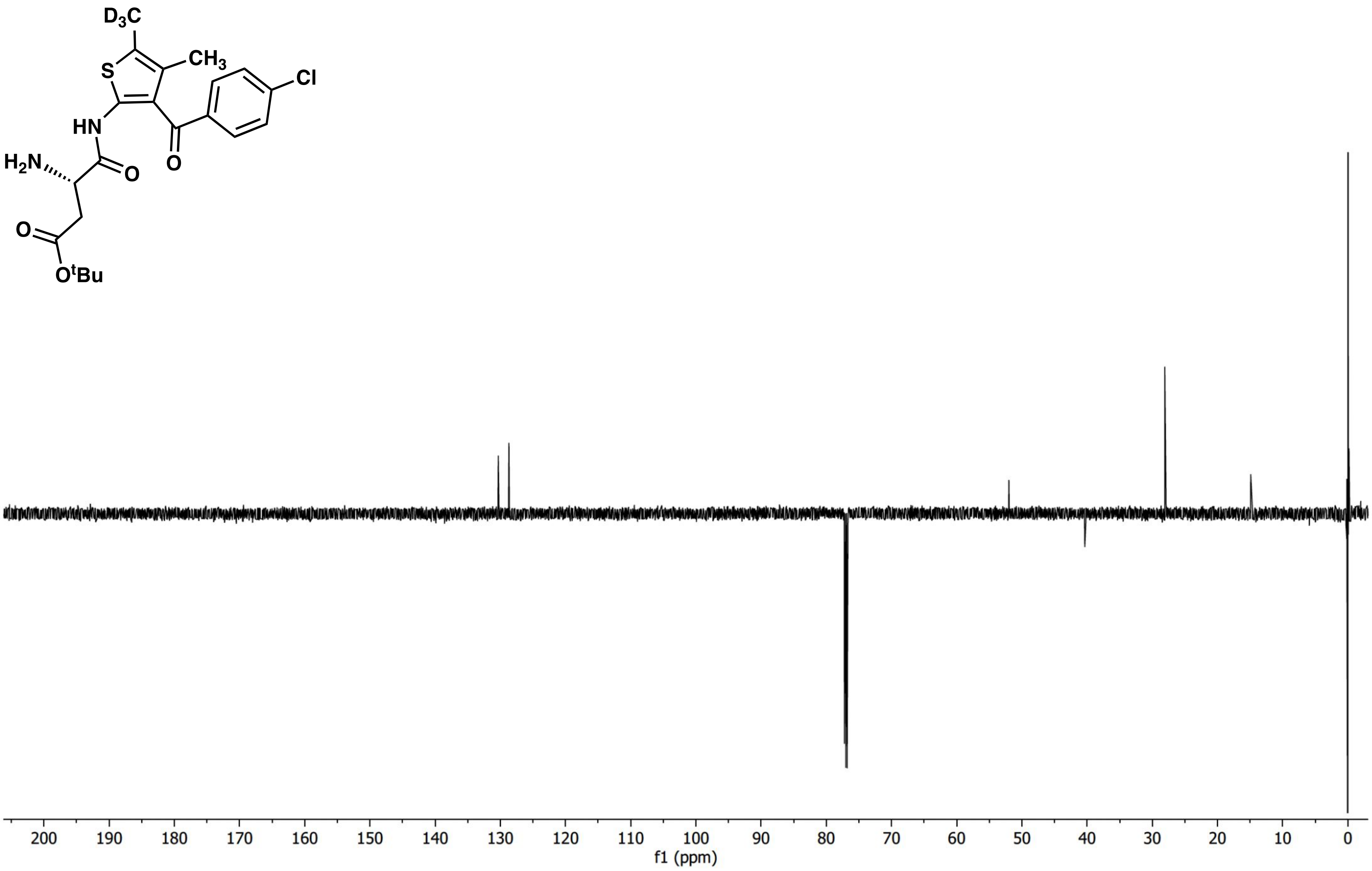

Figure S20. tert-butyl (3S)-3-amino-4-[[3-(4-chlorobenzoyl)-4-methyl-5-(trideuteriomethyl)-2-thienyl]amino]-4-oxo-butanoate (**12**) COSY NMR spectrum

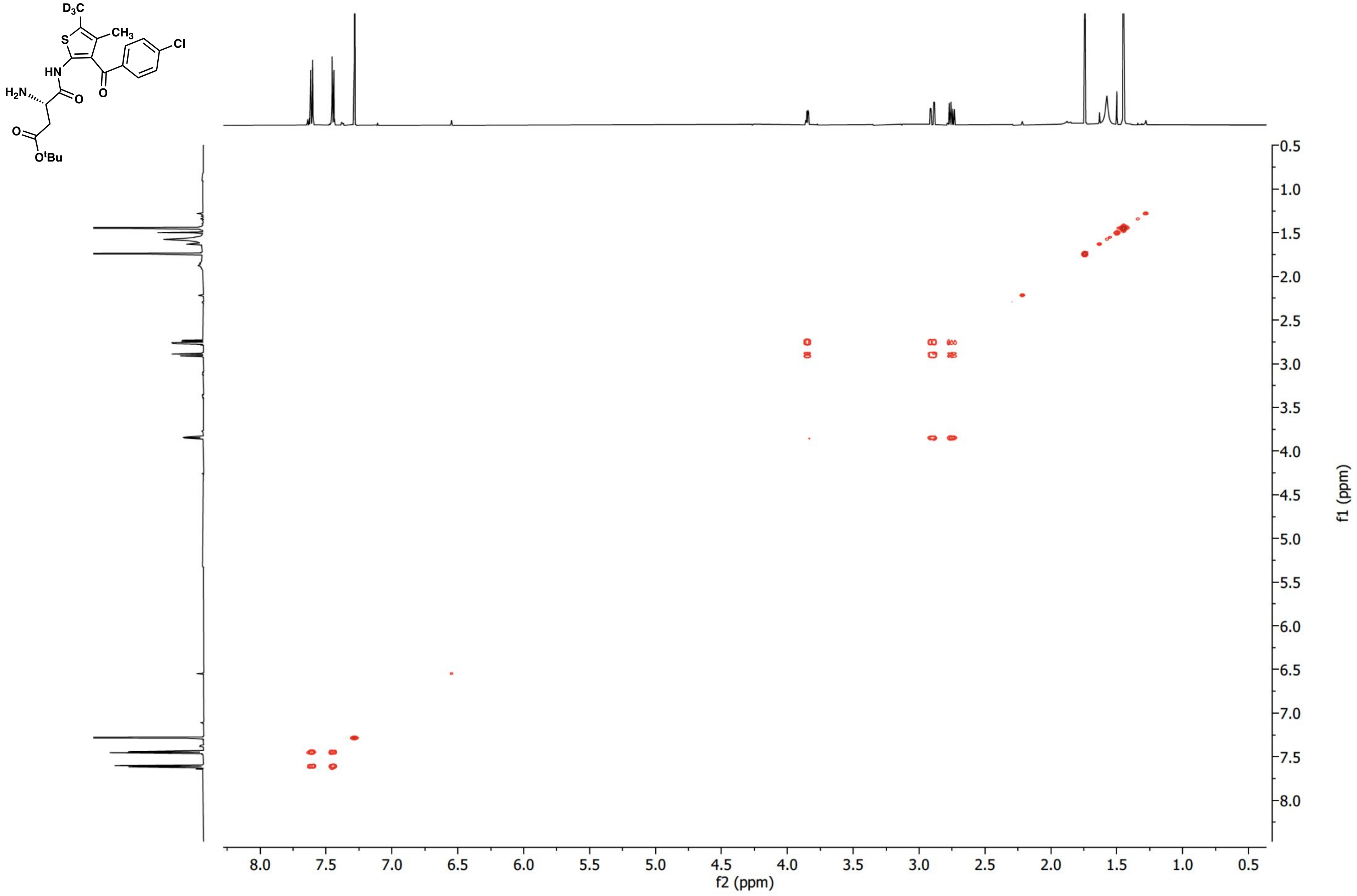

Figure S21. tert-butyl (3S)-3-amino-4-[[3-(4-chlorobenzoyl)-4-methyl-5-(trideuteriomethyl)-2-thienyl]amino]-4-oxo-butanoate (**12**) HSQC NMR spectrum

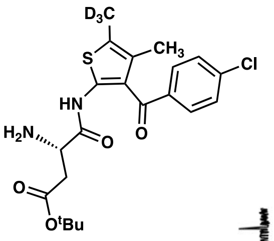

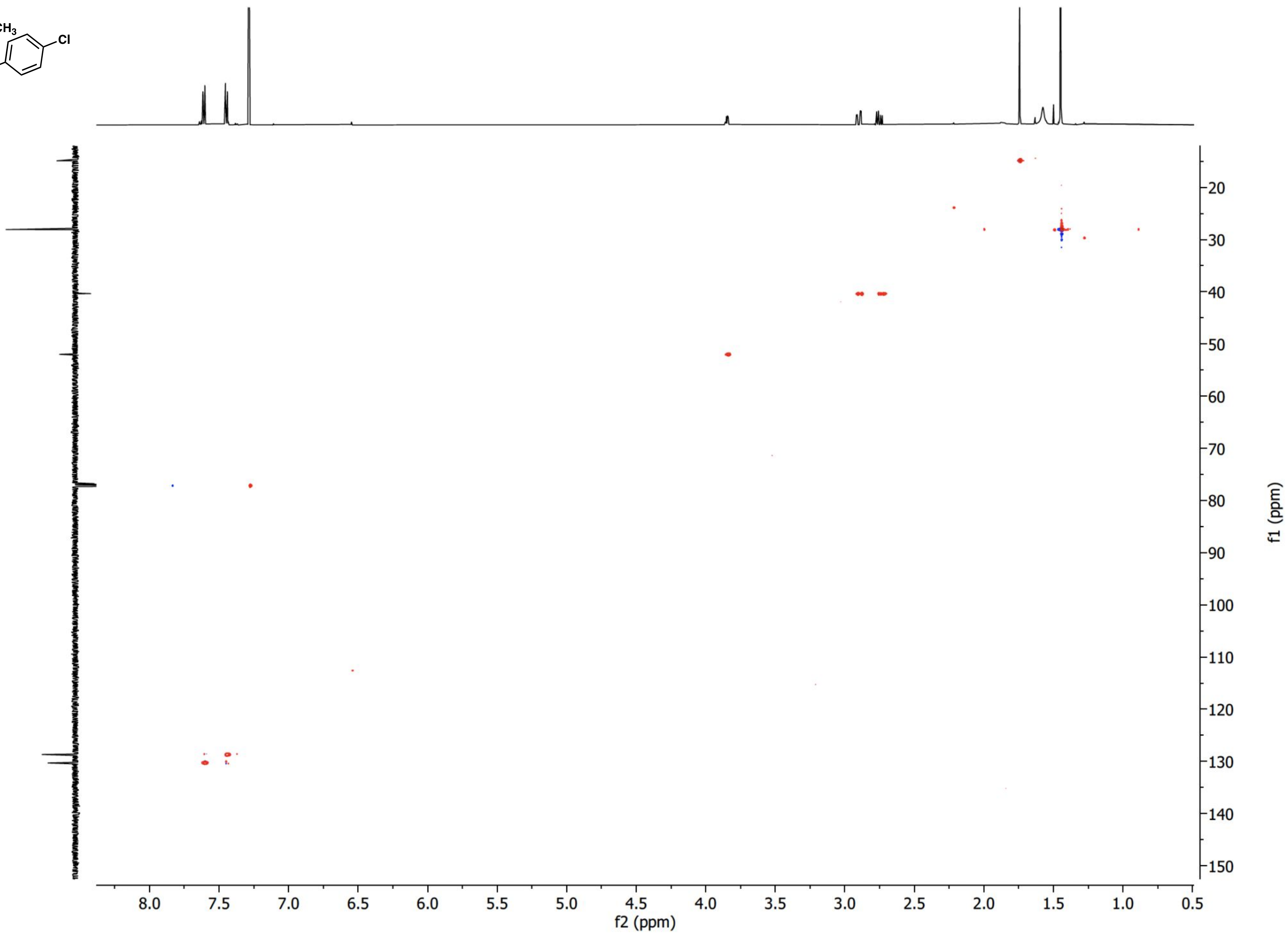

Figure S22. tert-butyl 2-[(3S)-5-(4-chlorophenyl)-6-methyl-2-oxo-7-(trideuteriomethyl)-1,3-dihydrothieno[2,3-e][1,4]diazepin-3-yl]acetate (**13**) 1H NMR spectrum [, 13C DEPTQ-135, , COSY, HMBC, HSQC].

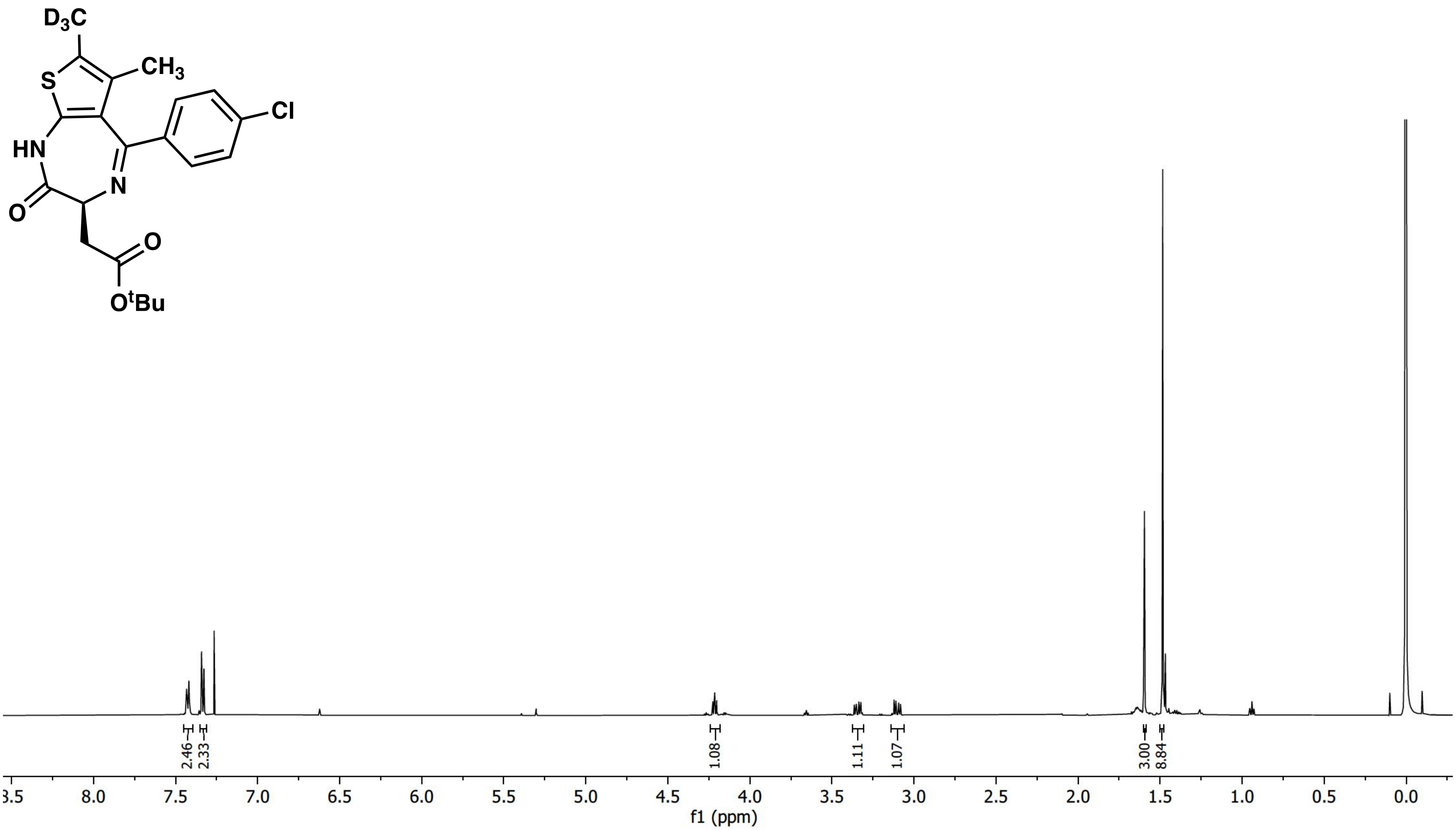

Figure S23. tert-butyl 2-[(3S)-5-(4-chlorophenyl)-6-methyl-2-oxo-7-(trideuteriomethyl)-1,3-dihydrothieno[2,3-e][1,4]diazepin-3-yl]acetate (**13**) 13C DEPTQ-135NMR spectrum

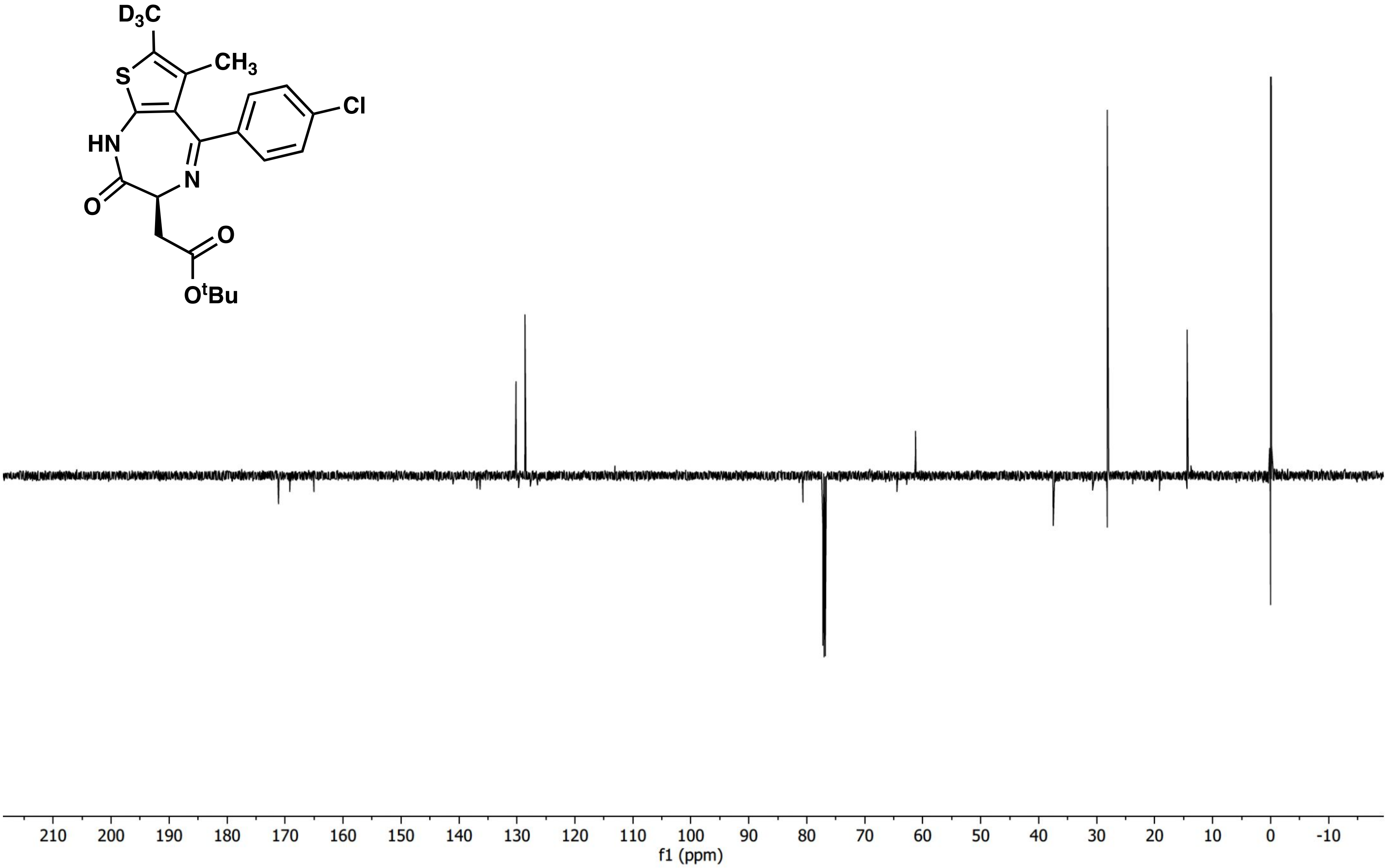

Figure S24. tert-butyl 2-[(3S)-5-(4-chlorophenyl)-6-methyl-2-oxo-7-(trideuteriomethyl)-1,3-dihydrothieno[2,3-e][1,4]diazepin-3-yl]acetate (**13**) COSY NMR spectrum

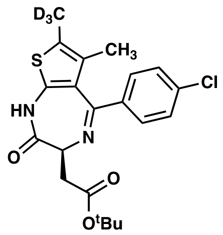

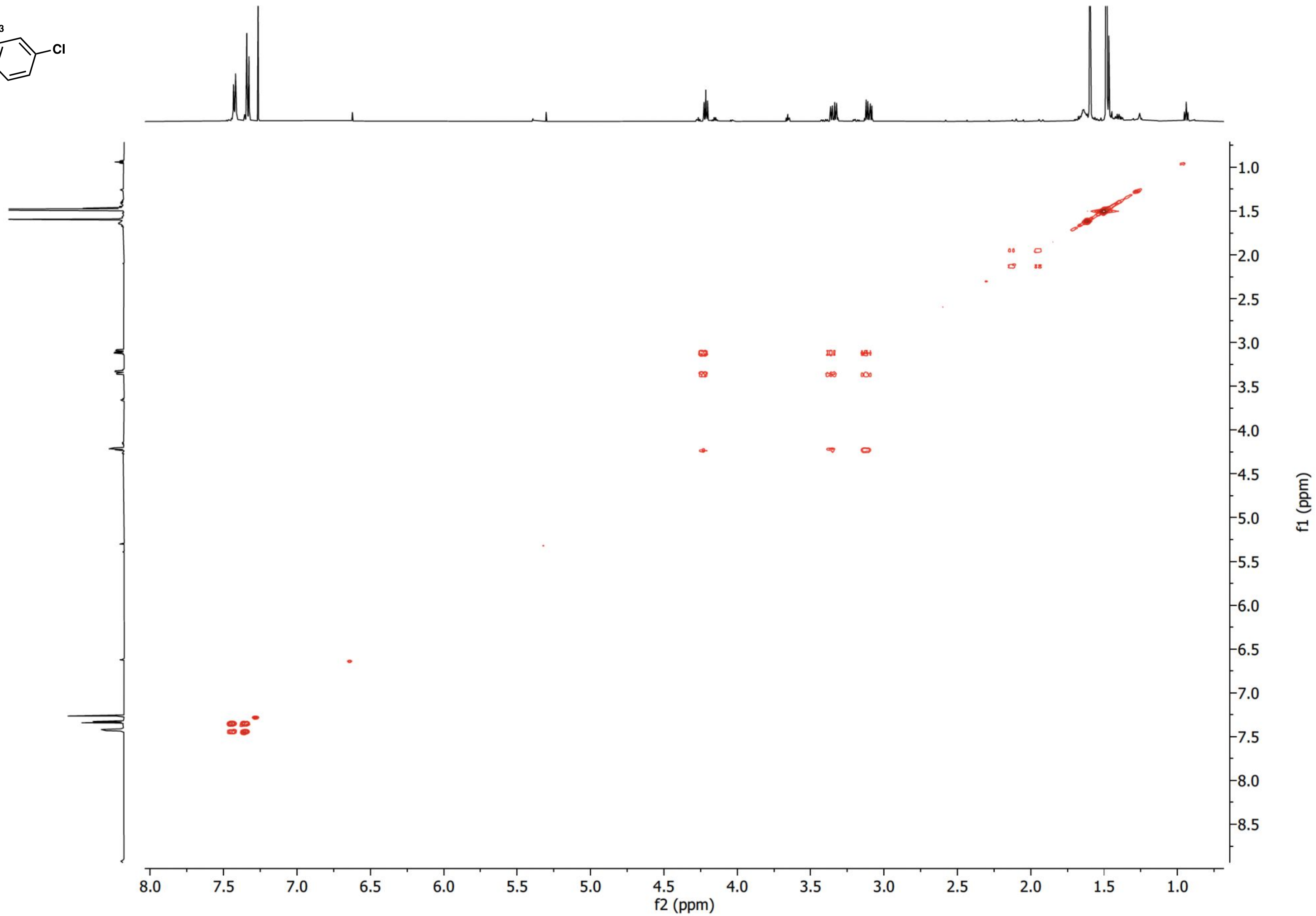

Figure S25. tert-butyl 2-[(3S)-5-(4-chlorophenyl)-6-methyl-2-oxo-7-(trideuteriomethyl)-1,3-dihydrothieno[2,3-e][1,4]diazepin-3-yl]acetate (**13**) HMBC NMR spectrum

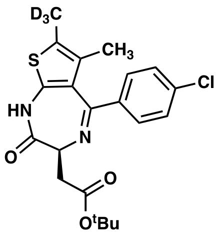

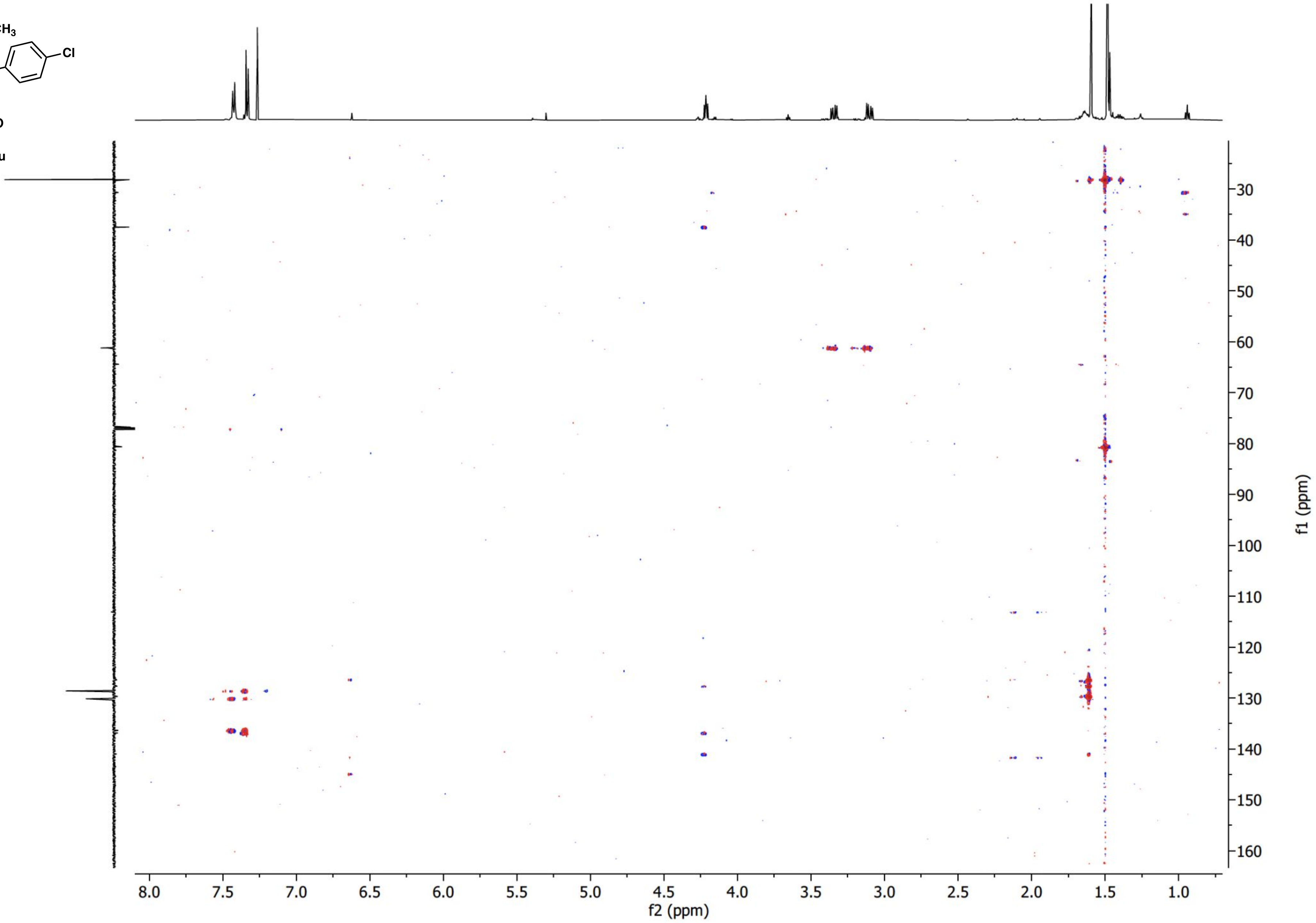

Figure S26. tert-butyl 2-[(3S)-5-(4-chlorophenyl)-6-methyl-2-oxo-7-(trideuteriomethyl)-1,3-dihydrothieno[2,3-e][1,4]diazepin-3-yl]acetate (**13**) HSQC NMR spectrum

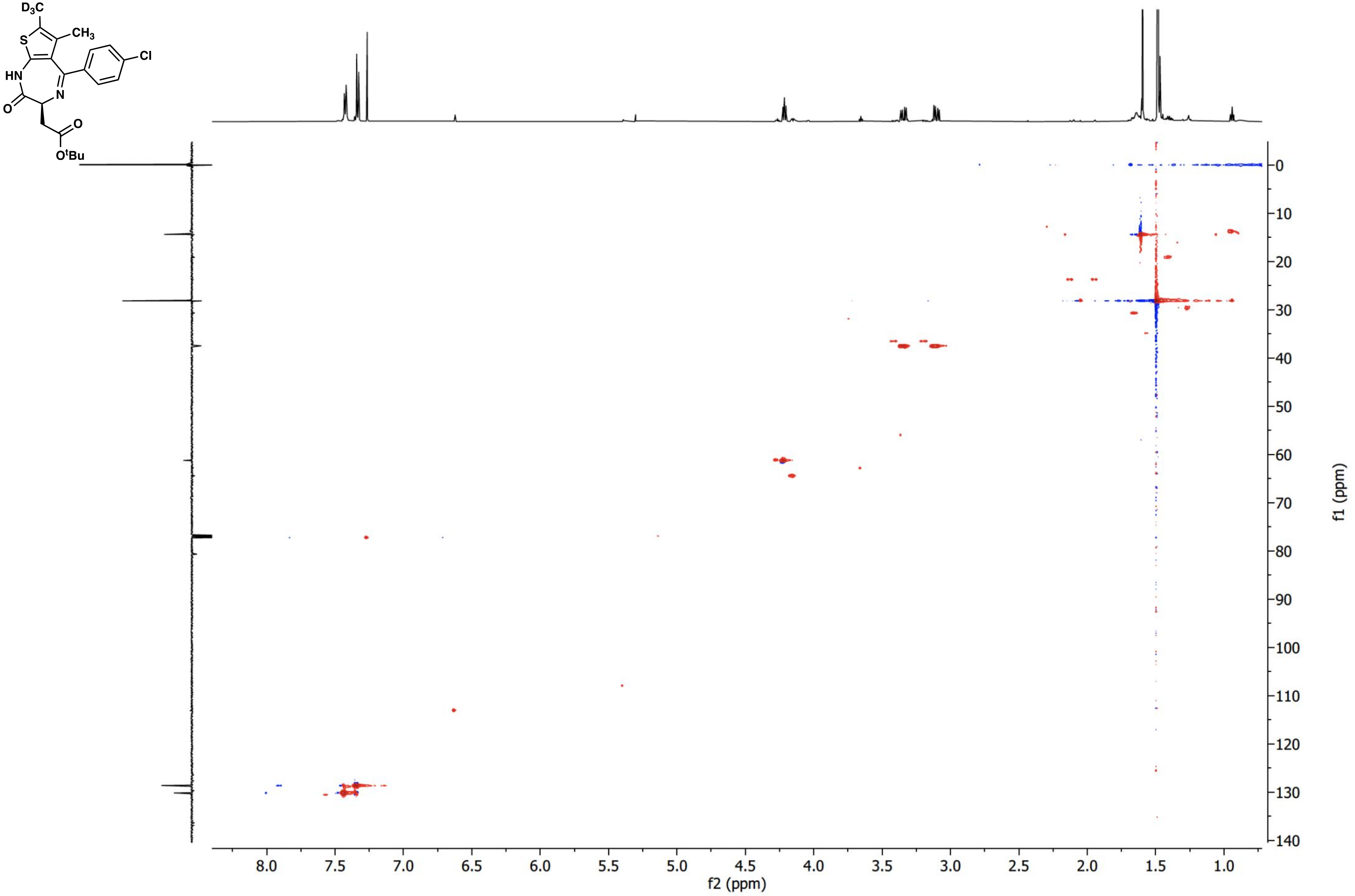

Figure S27. tert-butyl 2-[(9S)-7-(4-chlorophenyl)-5,13-dimethyl-4-(trideuteriomethyl)-3-thia-1,8,11,12-tetrazatricyclo[8.3.0.02,6]trideca-2(6),4,7,10,12-pentaen-9-yl]acetate, (+)-JQ1-D (**14**) 1H NMR spectrum

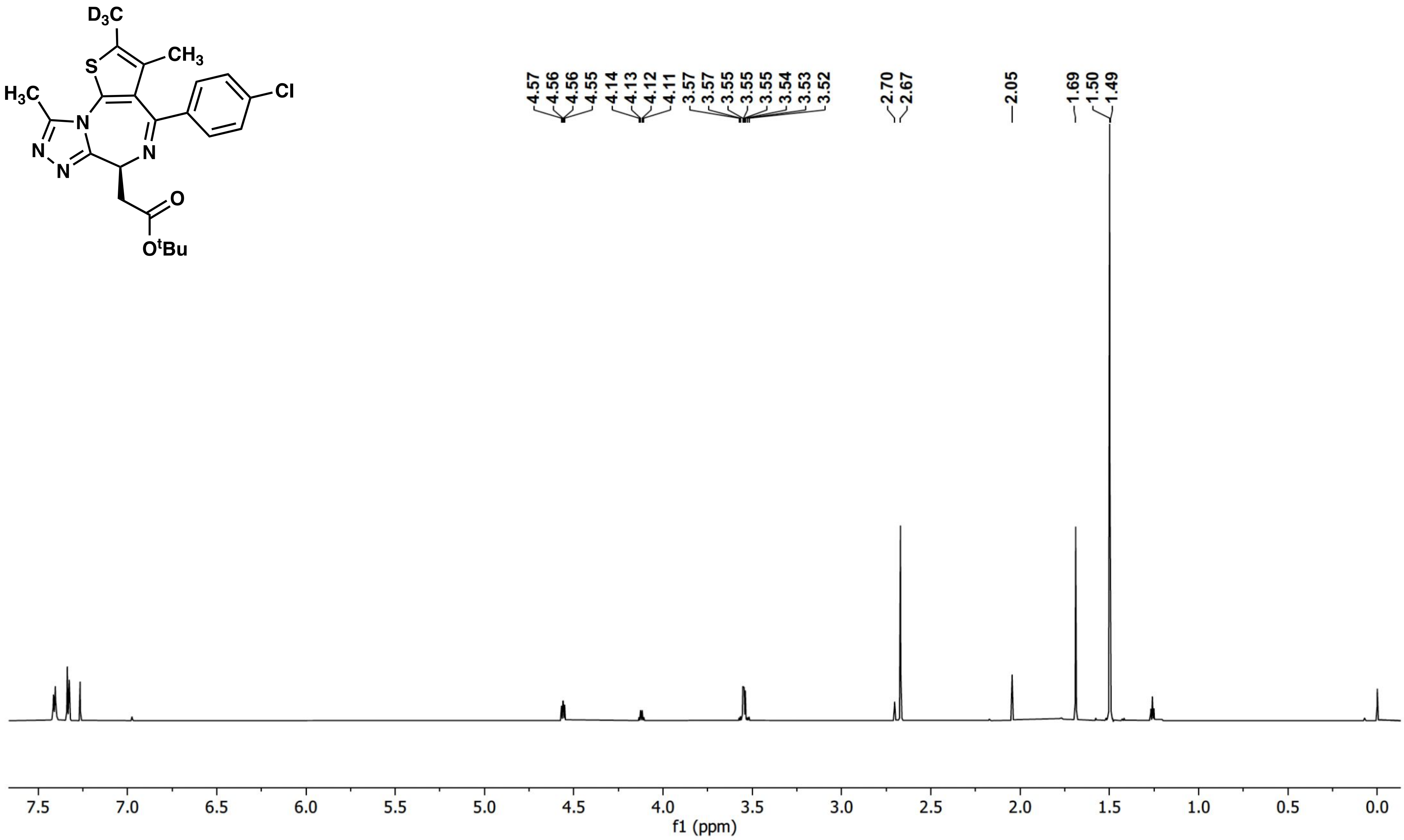

Figure S28. tert-butyl 2-[(9S)-7-(4-chlorophenyl)-5,13-dimethyl-4-(trideuteriomethyl)-3-thia-1,8,11,12-tetrazatricyclo[8.3.0.02,6]trideca-2(6),4,7,10,12-pentaen-9-yl]acetate, (+)-JQ1-D (**14**) 13C NMR spectrum

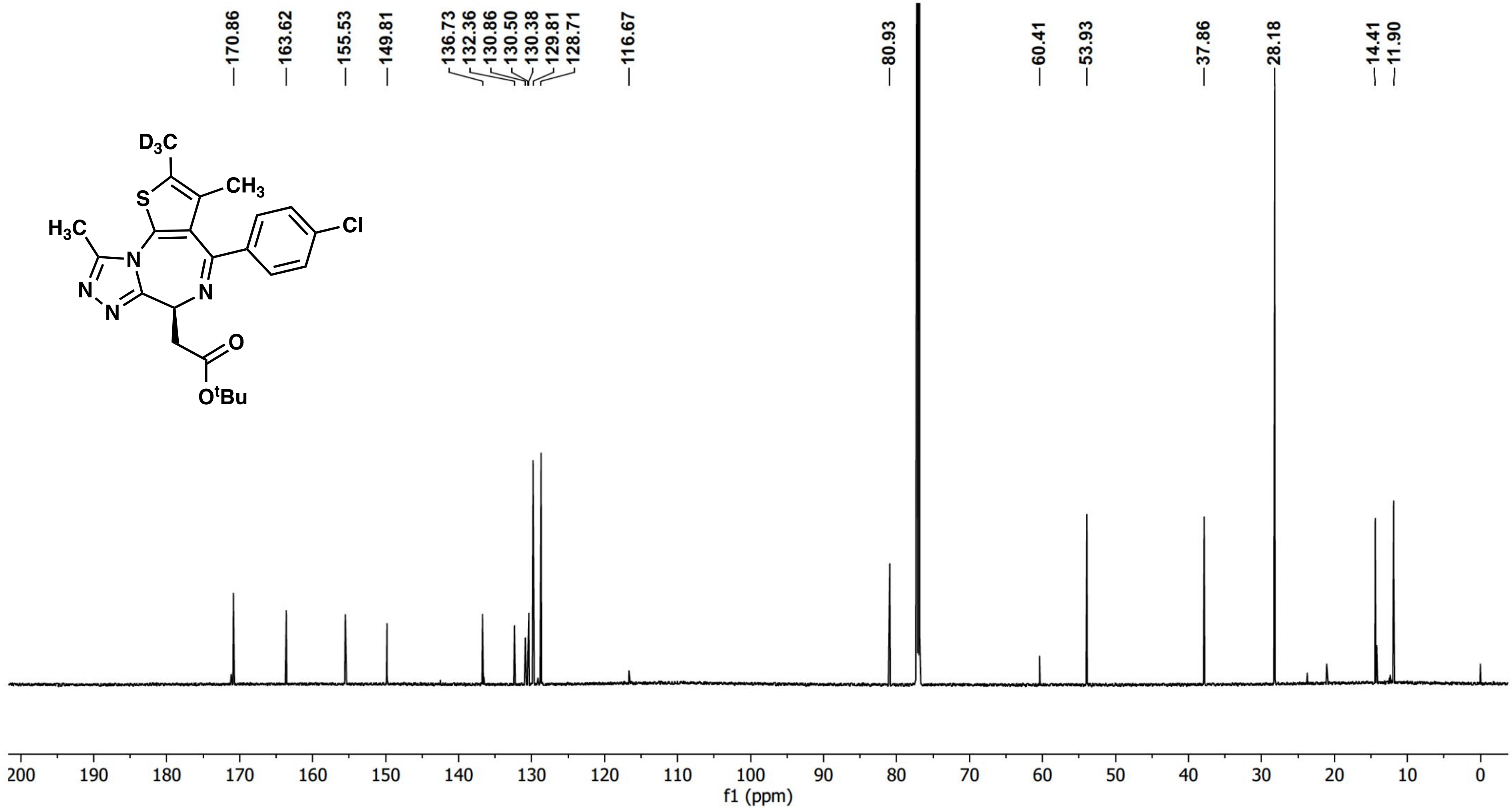

Supplement: Supplementary file 1 — ml3c00464_si_001.pdf [file ml3c00464_si_001.pdf]
